# Long-term intake of *Lactobacillus* *helveticus* enhances bioavailability of omega-3 fatty acids in the mouse retina

**DOI:** 10.1038/s41522-023-00474-5

**Published:** 2024-01-18

**Authors:** Pierre Lapaquette, Sébastien Terrat, Lil Proukhnitzky, Lucy Martine, Stéphane Grégoire, Bénédicte Buteau, Stéphanie Cabaret, Aurélie Rieu, Luis G. Bermúdez-Humarán, Pierre-Henry Gabrielle, Catherine Creuzot-Garcher, Olivier Berdeaux, Niyazi Acar, Marie-Agnès Bringer

**Affiliations:** 1 Univ. Bourgogne, UMR PAM A 02.102, Institut Agro Dijon, INRAE, F-21000 Dijon, France; 2 Agroécologie, Institut Agro, INRAE, Univ. Bourgogne, Univ. Bourgogne Franche-Comté, F-21000 Dijon, France; 3 Centre des Sciences du Goût et de l’Alimentation, CNRS, INRAE, Institut Agro, Université de Bourgogne, F-21000 Dijon, France; 4 ChemoSens Platform, Centre des Sciences du Goût et de l’Alimentation, CNRS, INRAE, Université Bourgogne Franche-Comté, Institut Agro; INRAE, PROBE Research infrastructure, ChemoSens facility, F-21000 Dijon, France; 5 Micalis Institute, Université Paris-Saclay, INRAE, AgroParisTech, F-78350 Jouy-en-Josas, France; 6 Department of Ophthalmology, University Hospital, F-21000 Dijon, France

**Keywords:** Microbiology, Health care

## Abstract

Omega-3 (n-3) polyunsaturated fatty acids (PUFAs), particularly docosahexaenoic acid (DHA), are required for the structure and function of the retina. Several observational studies indicate that consumption of a diet with relatively high levels of n-3 PUFAs, such as those provided by fish oils, has a protective effect against the development of age-related macular degeneration. Given the accumulating evidence showing the role of gut microbiota in regulating retinal physiology and host lipid metabolism, we evaluated the potential of long-term dietary supplementation with the Gram-positive bacterium *Lactobacillus helveticus* strain VEL12193 to modulate the retinal n-3 PUFA content. A set of complementary approaches was used to study the impact of such a supplementation on the gut microbiota and host lipid/fatty acid (FA) metabolism. *L. helveticus*-supplementation was associated with a decrease in retinal saturated FAs (SFAs) and monounsaturated FAs (MUFAs) as well as an increase in retinal n-3 and omega-6 (n-6) PUFAs. Interestingly, supplementation with *L. helveticus* enriched the retina in C22:5n-3 (docosapentaenoic acid, DPA), C22:6n-3 (DHA), C18:2n-6 (linoleic acid, LA) and C20:3n-6 (dihomo gamma-linolenic acid, DGLA). Long-term consumption of *L. helveticus* also modulated gut microbiota composition and some changes in OTUs abundance correlated with the retinal FA content. This study provides a proof of concept that targeting the gut microbiota could be an effective strategy to modulate the retinal FA content, including that of protective n-3 PUFAs, thus opening paths for the design of novel preventive and/or therapeutical strategies for retinopathies.

## Introduction

The retina is the tissue that lines the back of the eyes and converts light into electrical signals for the brain. It consists of the neuroretina that contains the light-sensitive cells, laying on the retinal pigmentary epithelium (RPE), a single layer of post-mitotic cells that nourishes and protects the neuroretina. The retina is the third tissue with the highest content in lipids in the human body after the adipose tissue and the brain. Retinal lipids are mostly phospholipids, representing 87.3% of total lipids in the neuroretina and 58.3% in the RPE^1,2^. This high content in phospholipids makes the retina very rich in fatty acids (FAs), particularly in docosahexaenoic acid (DHA) that is a polyunsaturated fatty acid (PUFA) belonging to the omega-3 (n-3) series^2^. DHA and its derivatives are crucial for visual function as well as for protecting retinal cell against inflammation, oxidative stress, apoptosis and neovascularization^3–7^. The essential role of n-3 PUFAs in the retina physiology is also supported by a number of observational studies indicating that a high dietary intake of fish rich in n-3 long chain (LC)-PUFAs is associated with a reduced risk of developing retinopathies such as age-related macular degeneration (AMD)^8^.

The retina physiology, including its FA composition, is very sensitive to diet^9–12^. In addition to impacting host lipids through the nature of the lipids it provides, diet can also indirectly influence host lipid metabolism by acting on the gut microbiota^13^. Indeed, the gut microbiota is involved in the regulation of different aspects of the host lipid metabolism (e.g., intestinal absorption, tissue storage, systemic transport and endogenous biosynthesis)^14–18^.

A growing body of evidence suggests the existence of a gut microbiota-retina axis. Alterations of the gut microbiota have been described in patients with retinal diseases, including AMD^19–21^. Moreover, several studies suggest that the gut microbiota could influence pathophysiological mechanisms in the retina such as neurodegeneration, pathological vascularization and inflammation^19,22–25^. The gut microbiota could also affect the retina lipid content. Comparison of the lipidome of retinas from germ-free mice and conventionally raised mice revealed that the presence of gut microbiota is associated with change in the glycerophospholipids profile of the retina^26,27^. Moreover, we recently reported that modulating gut microbiota composition through a prebiotic-based approach leads to alterations in liver FA content, which is known to address FA-rich lipoproteins to the retina^14,28^.

In light of the evidence supporting the existence of a gut microbiota-retina axis and the role of the gut microbiota in regulating host lipid metabolism, manipulating the gut microbiota to modulate retinal lipid content seems an attractive approach. In this line, the use of probiotics, defined as “live microorganisms that, when administered in adequate amounts, confer a health benefit on the host” could be an interesting strategy^29^. The lactic acid bacteria (LAB) are a group of microorganisms commonly used as probiotics. Interestingly, several experimental studies suggest that retinal physiology could be influenced by oral administration of probiotics^30–32^. In the present study, we investigated the impact of long-term dietary intake of a LAB strain, *Lactobacillus helveticus* (*L. helveticus*) VEL12193, on the bioavailability of FAs to the retina. This strain has been isolated from dairy products (i.e., cheese) and has been previously characterized for its immunomodulatory properties^33^. Mice were fed either a control diet or a diet enriched in *L. helveticus* for 6 months. Gut microbiota composition was analyzed, and host lipid metabolism was studied in different organs/tissues of interest (liver, plasma and retina).

## Results

### Body weight, food intake and fat deposition

**Fig. 1:**
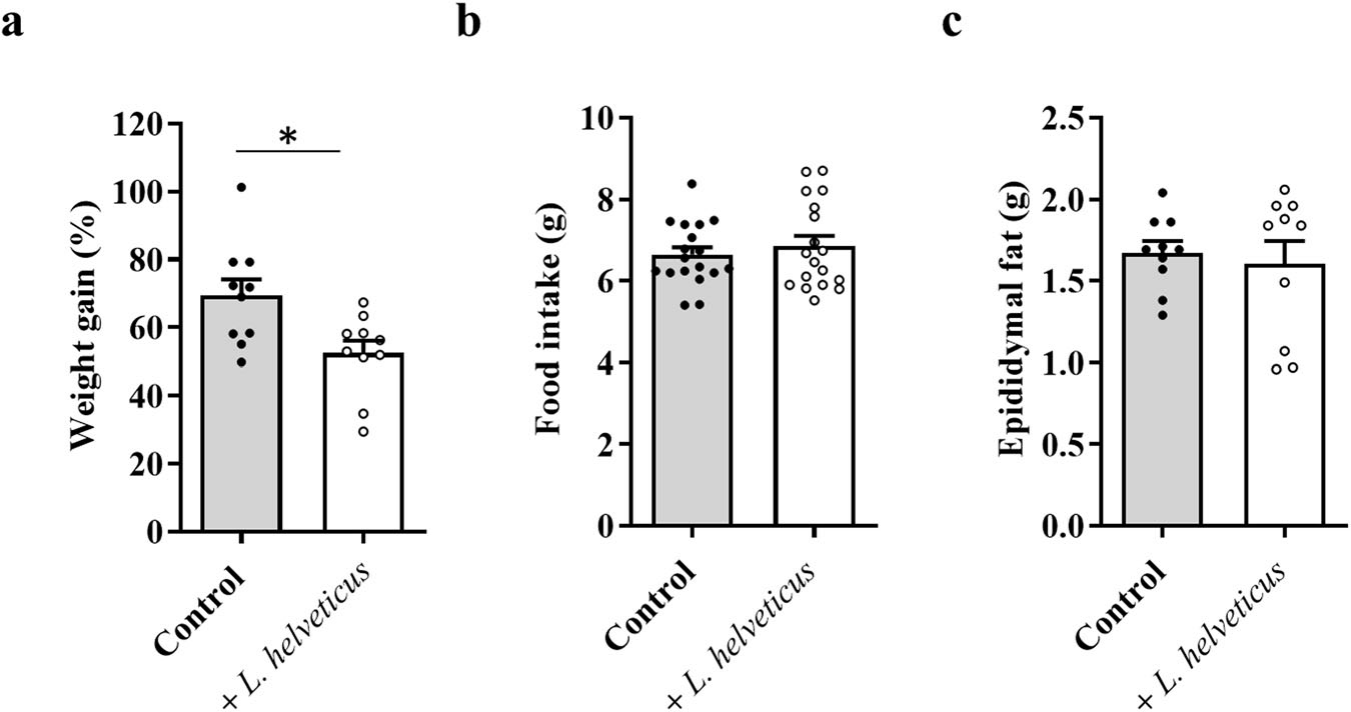
Effect of *L. helveticus* on weight gain, food intake and epididymal fat deposition. **a** Weight gain. Results are expressed as the percentage of weight gained after a 6 months period of exposure to a control diet or *L. helveticus*-supplemented diet. n = 10/group. **b** Food intake. Results are expressed as the weight (grams, g) of food consumed per day and per mouse. Nine independent measurements were performed per cage (2 cages/group). **c** Epididymal fat deposition. The epididymal adipose tissue from the left fat pad was weighted. Results are expressed in grams (g). n = 10/group. **a**–**c** Data are presented as mean ± s.e.m. Mann-Whitney test (* *p* < 0.05).

Weight gain was evaluated in mice after a 6-month exposure period to a diet supplemented or not with *L. helveticus* VEL12193. Administration of *L. helveticus* was well-tolerated by the mice, with no noticeable side effects, including on the consistency of feces. We observed that supplementating mice with *L. helveticus* significatively limited weight gain compared to control mice (Fig. 1a). This phenotype was not the consequence of a change in eating behavior since the amount of food consumed daily was identical for the two groups (Fig. 1b). It was also not associated with modification in visceral fat deposition, as evidenced by measurement of epidydimal fat weight (Fig. 1c).

### Lipid metabolism in the liver

**Fig. 2:**
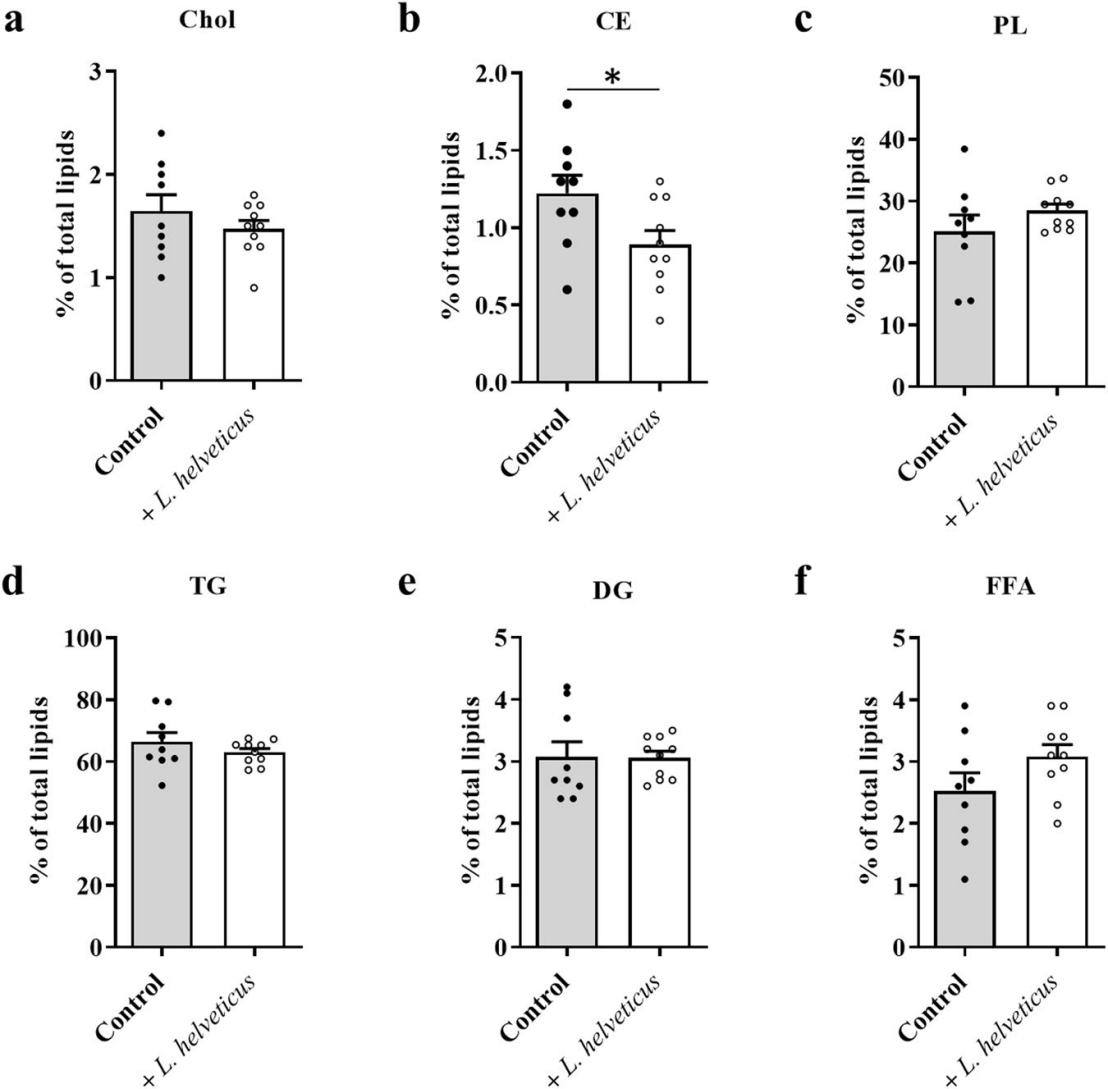
Effect of long-term consumption of *L. helveticus* on lipid classes in the liver. **a** Cholesterol (Chol). **b** Cholesteryl esters (CE). **c** Phospholipids (PL). **d** Triglycerides (TG). **e** Diglycerides (DG). **f** Free fatty acids (FFA). Results are expressed as abundance (%) relative to total lipids defined as 100%. **a**–**f** Control group, *n* = 9. *L. helveticus* group, *n* = 10. Data are presented as mean ± s.e.m. Mann-Whitney test (*p < 0.05).

We investigated whether long-term consumption of *L. helveticus* VEL12193 impacts lipids in the liver, a central organ of lipid metabolism. Analysis of the distribution of the lipids in the different classes showed that dietary supplementation with *L. helveticus* only significantly decreased the abundance of cholesteryl esters (CE; control group: 1.2% ± 0.1% and *L. helveticus* group: 0.9% ± 0.1% of total lipids; Fig. 2).

**Fig. 3:**
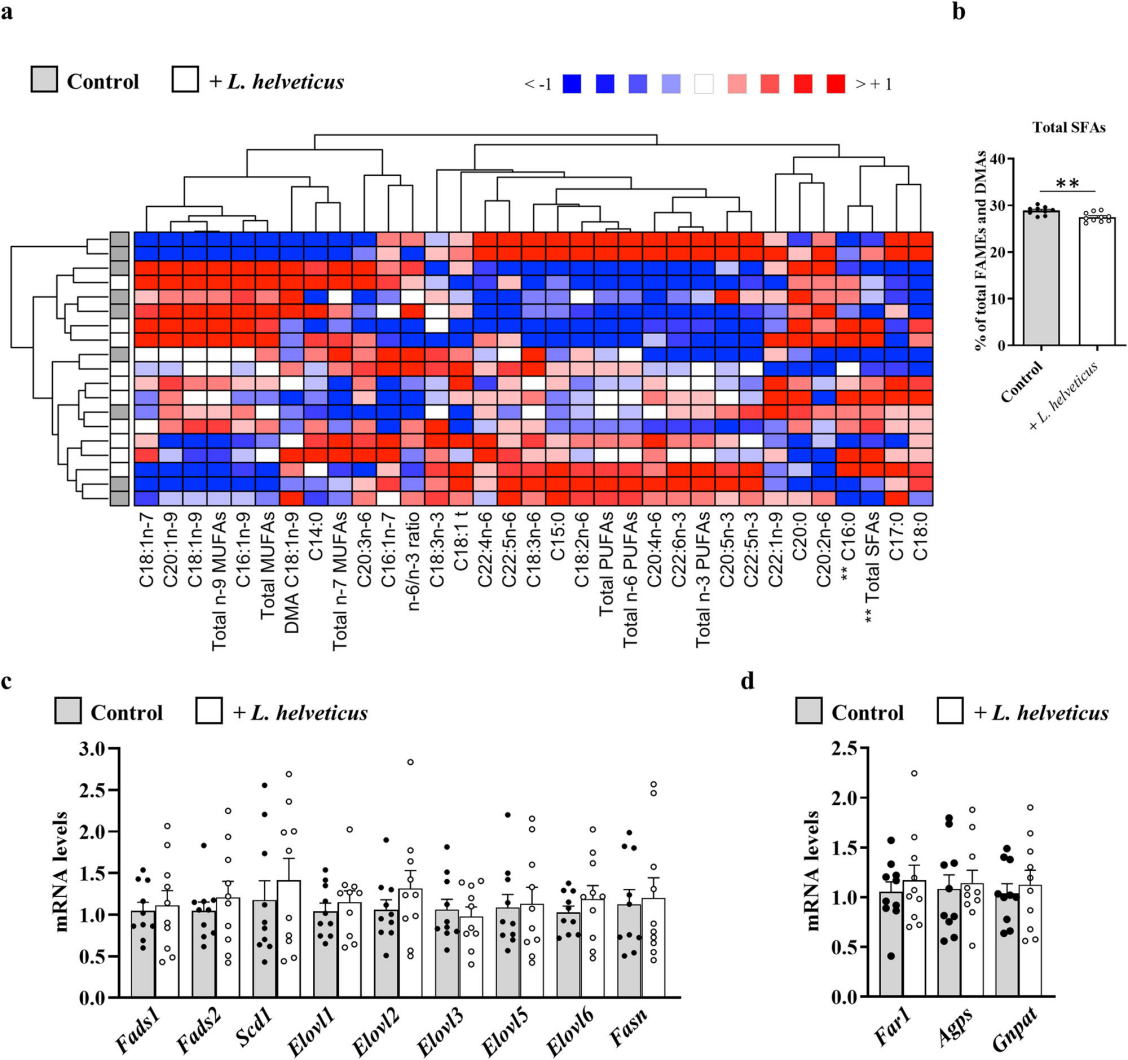
Fatty acid content in the liver of mice exposed lengthily to *L. helveticus*. **a** Heat map showing the hepatic abundance of each fatty acid methyl esters (FAME) or dimethylacetal (DMA) relative to total FAMEs + DMAs (defined as 100%) in mice fed a control diet or fed a diet supplemented with *L. helveticus*. The ratio of total n-6 PUFAs/total n-3 PUFAs was calculated (n-6/n-3 ratio). Samples from the control group are in gray, and those from the group supplemented with *L. helveticus* are in white. **b** Percentages of total SFAs relative to total FAMEs + DMAs (defined as 100%) in mice fed a control diet or fed a diet supplemented with *L. helveticus*. **c** Hepatic expression of genes encoding enzymes involved in the biosynthesis of fatty acids: acyl-CoA (8-3)-desaturase (*Fads1*), acyl-CoA 6-desaturase (*Fads2*), acyl-CoA desaturase 1 (*Scd1*), elongation of very long chain fatty acids proteins 1, 2, 3, 5 and 6 (*Elovl1*, *Elovl2*, *Elovl3*, *Elovl5* and *Elovl6*), and fatty acid synthase (Fasn). **d** Hepatic expression of genes encoding enzymes involved in the biosynthesis of plasmalogens: fatty acyl-CoA reductase 1 (*Far1*), alkylglycerone-phosphate synthase (*Agps*) and dihydroxyacetone phosphate acyltransferase (*Gnpat*). The levels of mRNA were normalized to *Hprt* mRNA level for calculation of the relative levels of transcripts. mRNA levels are illustrated as fold change. Lipid analyzes (**a** and **b**): *n* = 9 for the control group and *n* = 10 for the *L. helveticus* group. Gene expression (**c** and **d**): *n* = 10/group. **b**–**d** Data are presented as mean ± s.e.m. Mann-Whitney test (**p < 0.001).

As changes in the composition of the gut microbiota can affect the metabolism of hepatic FAs^14,17^, the FA profile and the expression level of a set of genes involved in FA biosynthesis were analyzed in the liver of mice fed *L. helveticus*-supplemented diet (Fig. 3 and Supplementary Table 1). Dietary supplementation with *L. helveticus* had little effect on liver FA content. A significant decrease in the hepatic abundance of total SFAs, which probably ensues from the significant reduction of the main SFA species (C16:0, palmitic acid), was observed in *L. helveticus*-treated mice compared to control mice (Fig. 3a and b, and Supplementary Table 1). The decrease in C16:0 was not associated with a modulation of the expression of *Fasn*, which encodes FAS, an enzyme involved in de novo lipogenesis from simple precursors and whose primary product is C16:0 (Fig. 3c). Besides, no effect of *L. helveticus* was observed neither on MUFAs nor on PUFAs levels (Fig. 3a and Supplementary Table 1). Moreover, no modification of the expression level of genes coding for desaturases (*Fads1*, *Fads2* and *Scd1*), elongases (*Elovl1*, *Elovl2*, *Elovl3*, *Elovl5* and *Elovl6*) and plasmalogen biosynthesis (*Far1*, *Agps* and *Gnpat*) was observed (Fig. 3c and d).

Taken together, these results suggest that long-term exposure to *L. helveticus* only affects SFAs in the liver and that this phenotype is not related to a modulation of the hepatic expression level of enzymes involved in their biosynthesis.

### Circulating lipids

**Fig. 4:**
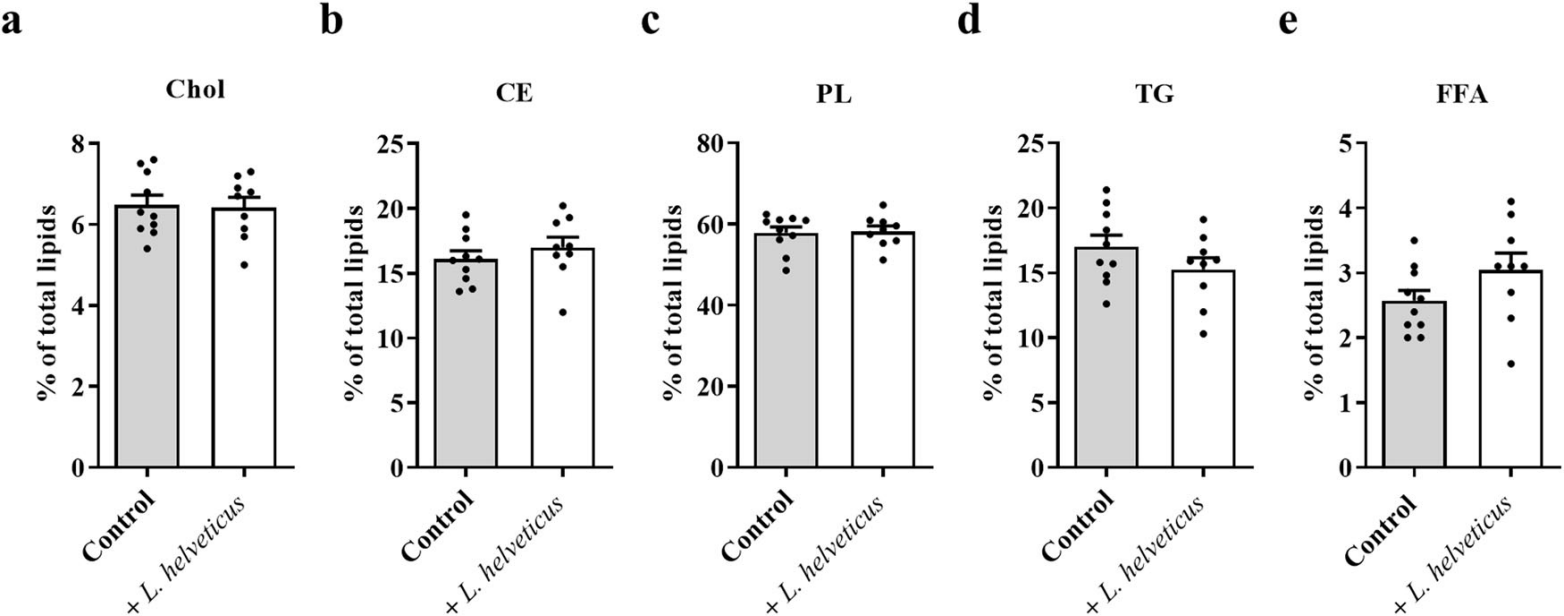
Effect of *L. helveticus* on lipid class distribution in the plasma. **a** Cholesterol (Chol). **b** Cholesteryl esters (CE). **c** Phospholipids (PL). **d** Triglycerides (TG). **e** Free fatty acids (FFA). Results are expressed as abundance (%) relative to total lipids defined as 100%. **a**–**e** Control group, *n* = 10. *L. helveticus* group, *n* = 9. Data are presented as mean ± s.e.m.

**Fig. 5:**
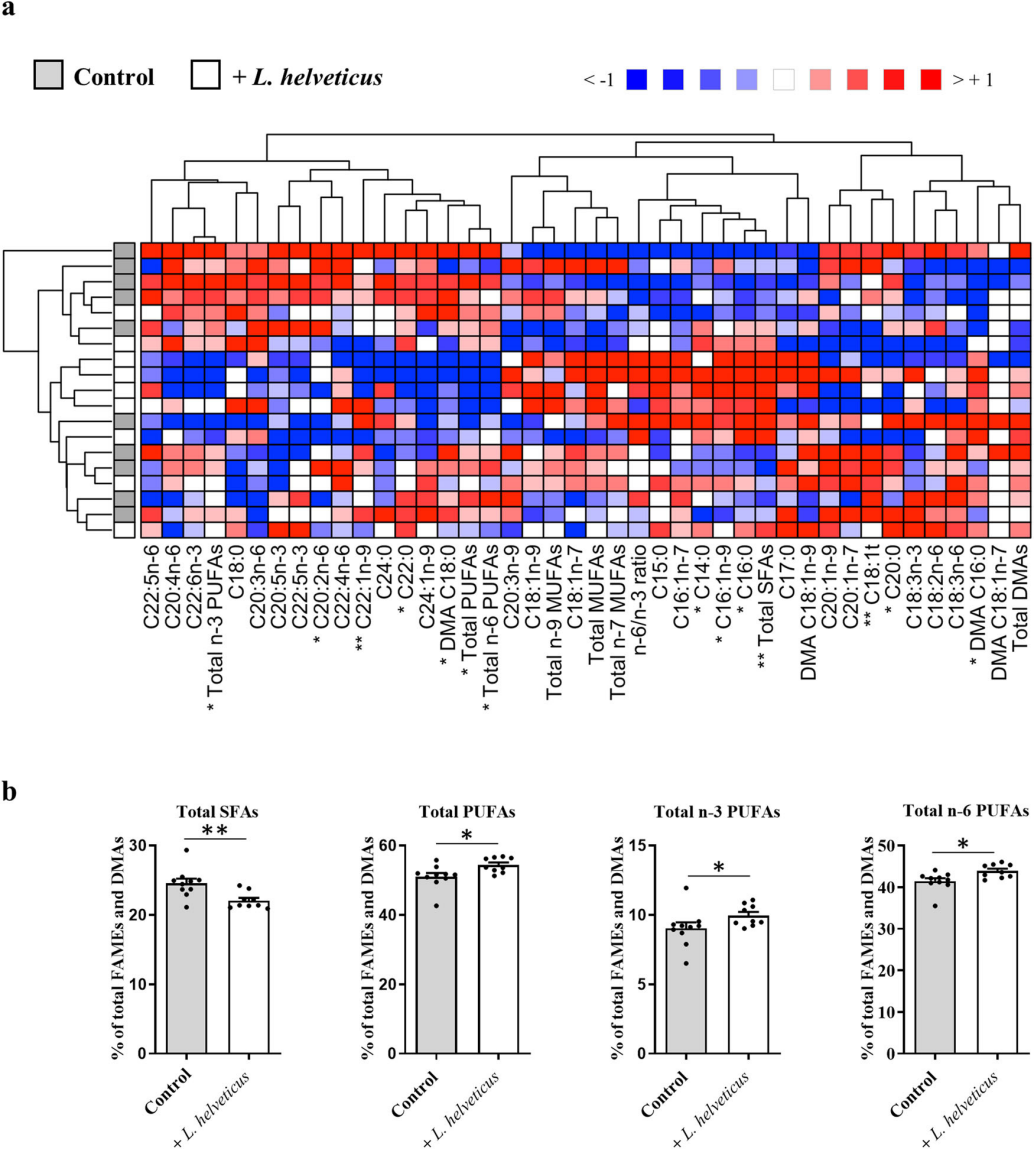
Fatty acid content in the plasma of mice lengthily exposed to *L. helveticus*. **a** Heat map showing the plasma abundance of each FAME or DMA relative to total FAMEs + DMAs (defined as 100%) in mice fed a control diet or fed a diet supplemented with *L. helveticus*. The ratio of total n-6 PUFAs/total n-3 PUFAs was calculated (n-6/n-3 ratio). Samples from the control group are in gray, and those from the group supplemented with *L. helveticus* are in white. **b** Percentages of total SFAs, total PUFAs, total n-3 PUFAs, and total n-6 PUFAs relative to total FAMEs + DMAs (defined as 100%) in mice fed a control diet or fed a diet supplemented with *L. helveticus*. Data are presented as mean ± s.e.m. **a**, **b** Control group, *n* = 10. *L. helveticus* group, *n* = 9. Mann-Whitney test (*p < 0.05 and **p < 0.01).

As plasma is the fluid that supplies FAs to the organs through lipoproteins, we analyzed the impact of long-term consumption of *L. helveticus* on the relative abundance of plasma lipid classes and FAs (Figs. 4, 5 and Supplementary Table 2). Plasma levels of cholesterol, cholesteryl esters, phospholipids, triglycerides and free FAs were similar in mice fed a diet supplemented with *L. helveticus* when compared to those measured in mice fed a control diet (Fig. 4). However, some changes were observed in the abundance of plasma FAs in *L. helveticus*-supplemented mice (Fig. 5). They were characterized by a significant decrease in the amounts of total SFAs, C14:0, C16:0 and C16:1n-9, and by a significant increase in the amounts of C20:0, C22:0, C22:1n-9, total PUFAs, total PUFAs n-3, total PUFAs n-6 and C20:2n-6 (Fig. 5a and b, and Supplementary Table 2).

In addition to fatty methyl esters (FAMEs), GC-FID enables the detection of dimethyl acetals (DMAs) that result from the acid-catalyzed transmethylation of the aldehyde aliphatic groups from the sn-1 position of plasmalogens, a specific class of glycerophospholipids^34^. Modifications in the distribution of DMAs species were observed in the plasma of *L. helveticus*-supplemented mice compared to control mice. Indeed, the amount of DMA C16:0 was decreased and that of DMA C18:0 was increased (Fig. 5a and Supplementary Table 2).

These results indicate that long-term exposure to *L. helveticus* is associated with remarkable changes in the plasma FA content.

### Lipid profile and metabolism in the retina

**Fig. 6:**
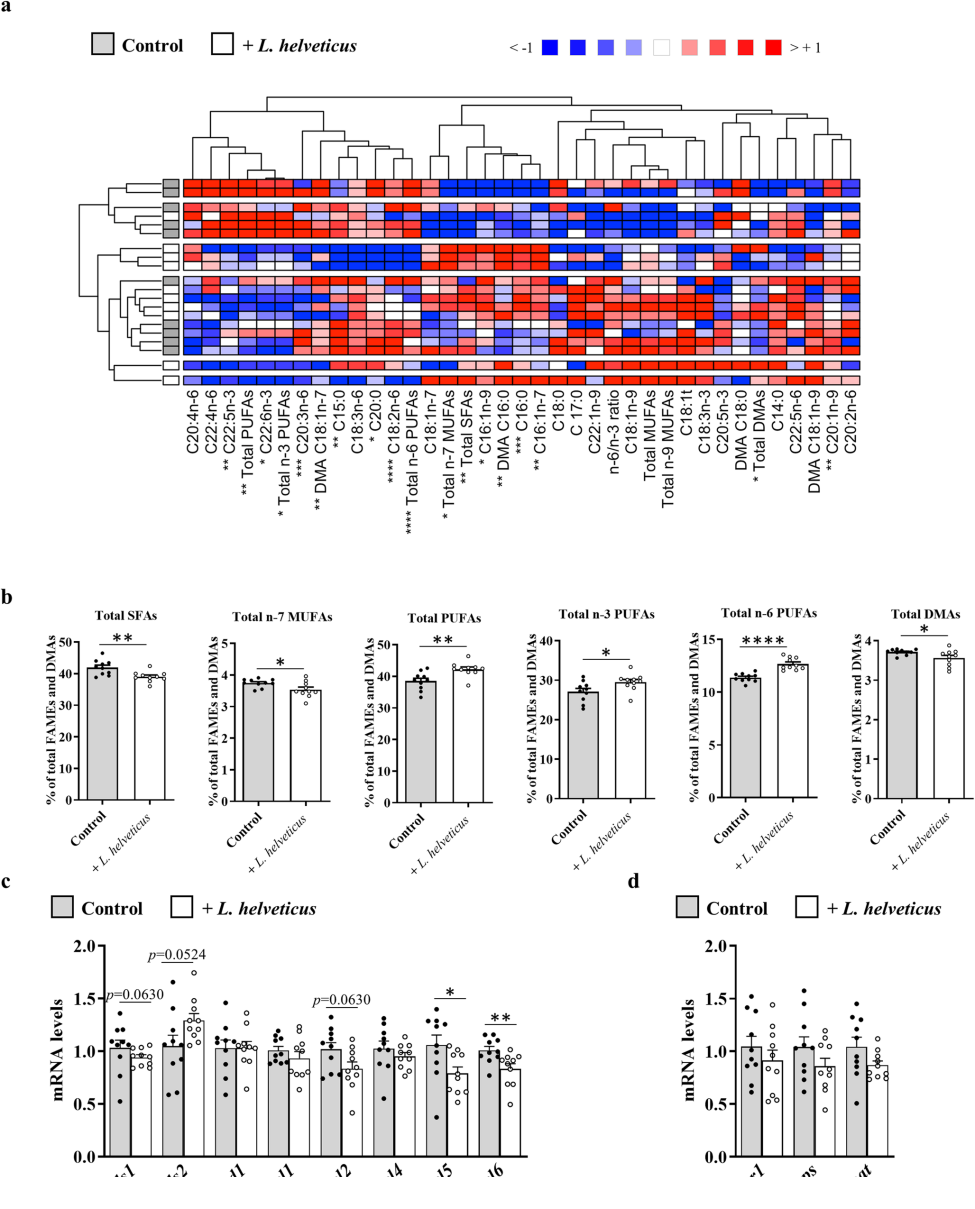
Fatty acid content in the retina of mice lengthily exposed to *L. helveticus*. **a** Heat map showing the retinal abundance of each FAME or DMA relative to total FAMEs + DMAs (defined as 100%) in mice fed a control diet or fed a diet supplemented with L. helveticus. The ratio of total n-6 PUFAs/total n-3 PUFAs was calculated (n-6/n-3 ratio). Samples from the control group are in gray, and those from the group supplemented with *L. helveticus* are in white. **b** Percentages of total SFAs, total n-7 MUFAs, total PUFAs, total n-3 PUFAs, total n-6 PUFAs and total DMAs relative to total FAMEs + DMAs (defined as 100%) in mice fed a control diet or fed a diet supplemented with *L. helveticus*. **c** Retinal expression of genes involved encoding enzymes involved in the biosynthesis of fatty acids: acyl-CoA (8-3)-desaturase (*Fads1*) and acyl-CoA 6-desaturase (*Fads2*), acyl-CoA desaturase 1 (*Scd1*), and elongation of very long chain fatty acids proteins 1, 2, 4, 5 and 6 (*Elovl1*, *Elovl2*, *Elovl4*, *Elovl5* and *Elovl6*). **d** Hepatic expression of genes encoding enzymes involved in the biosynthesis of plasmalogens: fatty acyl-CoA reductase 1 (*Far1*), alkylglycerone-phosphate synthase (*Agps*) and dihydroxyacetone phosphate acyltransferase (*Gnpat*). The levels of mRNA were normalized to *Hprt* mRNA level for calculation of the relative levels of transcripts. mRNA levels are illustrated as fold change. (**a**–**d**) *n* = 10/group. (**b**–**d**) Data are presented as mean ± s.e.m. Mann-Whitney test (*p < 0.05, **p < 0.01, ***p < 0.001 and ****p < 0.0001).

Analysis of the retinal FA content revealed profound changes in mice fed a *L. helveticus*-supplemented diet (Fig. 6 and Supplementary Table 3). As observed in liver and plasma, *L. helveticus* consumption was associated with a significant decrease in the amount of total SFAs in the retina that may result from the decrease in C16:0 (Fig. 6a and b, and Supplementary Table 3). The retina of the *L. helveticus* group of mice also exhibited a reduced amount of total n-7 MUFAs, C16:1n-7 and of two MUFAs of the n-9 series (C16:1n-9 and C20:1n-9) compared to control mice (Fig. 6a and b, and Supplementary Table 3). These changes in SFAs and MUFAs levels were balanced by an enrichment of the retina in PUFAs from both the n-6 and n-3 series (Fig. 6a and b, and Supplementary Table 3). Particularly, *L. helveticus* promoted a significant enrichment of the retina in C22:5n-3 (n-3 docosapentaenoic acid, n-3 DPA), C22:6n-3 (docosahexaenoic acid, DHA), C18:2n-6 (linoleic acid, LA) and C20:3n-6 (dihomo gamma-linolenic acid, DGLA) (Fig. 6a and Supplementary Table 3). We investigated whether changes in the retinal FA content could be associated with modulation of the expression of enzymes involved in their biosynthesis (Fig. 6c). Unexpectedly, retinal expression of the gene encoding the elongase ELOVL5, involved in the elongation of PUFAs to LC-PUFAs, was significantly decreased in mice fed a *L. helveticus*-supplemented diet (Fig. 6c).

In the retina, FAs are almost exclusively esterified on phospholipids. In order to find out accurately which phospholipid species were affected by the FA changes occurring in the retina, an HPLC-MS analysis was performed (Supplementary Table 4). We identified 128 phospholipid species, including 26 phosphatidylethanolamine (PE) species, 18 plasmenylethanolamine (PlE) species, 35 phosphatidylcholine (PC) species, 3 plasmenylcholine (PlC) species, 12 phosphatidylserine (PS) species, 1 plasmenylserine (PlS) species, 19 phosphatidylinositol (PI) species and 14 sphingomelin (SM) species (Supplementary Table 4). No change in the abundance of SMs was observed in the retina of mice fed a *L. helveticus*-supplemented diet when compared to control mice. However, the abundance of 5 PEs, 4 PCs and 4 PIs was significantly increased in the mouse retina as a consequence of long-term consumption of *L. helveticus* (Supplementary Table 4). Interestingly, among these species, the probiotic increased the relative abundance of two PE species esterified with DHA at the sn-2 position (namely PE(16:0/22:6), which is the second most abundant PE species in the retina, and PE(16:1/22:6)). In addition, a significant decrease in the abundance of the main PI species, PI(18:0/20:4), was observed in the retina of the *L. helveticus* group (Supplementary Table 4). The relative abundance of 5 PlEs was increased in *L. helveticus*-supplemented mice (Supplementary Table 4), but these changes were not associated with a modification in the expression level of genes encoding enzymes involved in Pls biosynthesis (Fig. 6d).

Altogether, these results showed that long-term consumption of *L. helveticus* modulates the retinal FA content and, particularly, enriches this tissue in PUFAs that are known to have beneficial properties for retinal health.

### Impact of long-term consumption of *L. helveticus* on gut microbiota communities

**Fig. 7:**
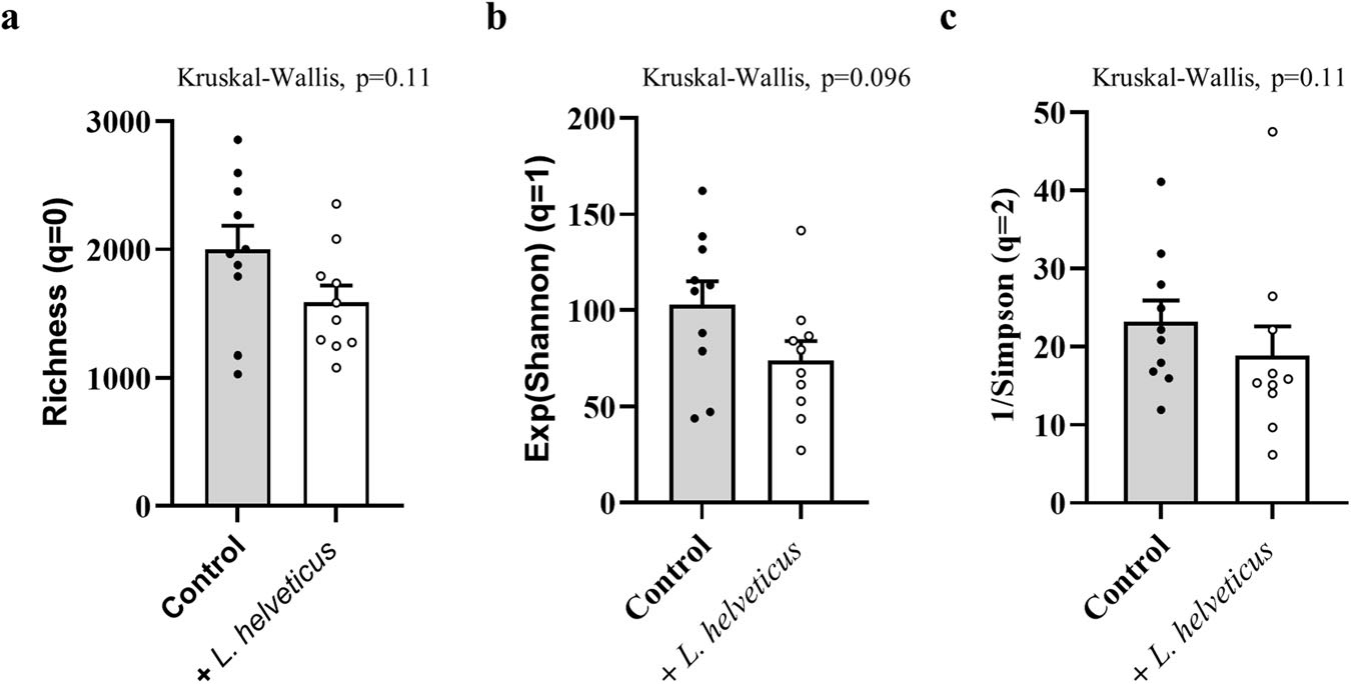
Hill’s diversity of the gut microbiota in *L. helveticus*-supplemented mice based on OTUs determination after ReClustOR refining. **a** *q* = 0 (species richness). **b** q = 1 (exponential of Shannon entropy). **c** *q* = 2 (reciprocal of Simpson index). **a**–**c** *n* = 10/group. Data are presented as mean ± s.e.m.

Since *L. helveticus* might indirectly affects host lipids by modulating the resident gut microbiota, we evaluated whether long-term consumption of *L. helveticus* has impacted its composition. Comparison of the fecal microbiota between mice fed a *L. helveticus*-supplemented diet and control mice showed no significant difference regarding the Hill’s diversity indices, indicating that this long-term bacterial supplementation did not affect the gut microbiota alpha-diversity (Fig. 7). The relative abundance of the major phyla and genera were also similar in the fecal microbiota of control mice and *L. helveticus*-supplemented mice (Supplementary Figs. 1a and 2a). Non-metric multidimensional scaling (NMDS) ordination of communities at the phylum level or at the genus level did not reveal any different microbial clustering between control mice and *L. helveticus*-supplemented mice (Supplementary Figs. 1b and 2b). In addition, PERMANOVA analysis were not significantly different between control mice and *L. helveticus*-supplemented mice showing that the overall distributions and abundances of phyla and genera were similar in the two groups (Supplementary Figs. 1b and 2b). Altogether, these results indicated that long-term consumption of *L. helveticus* did not markedly altered the initial composition of the resident gut microbiota in mice.

**Fig. 8:**
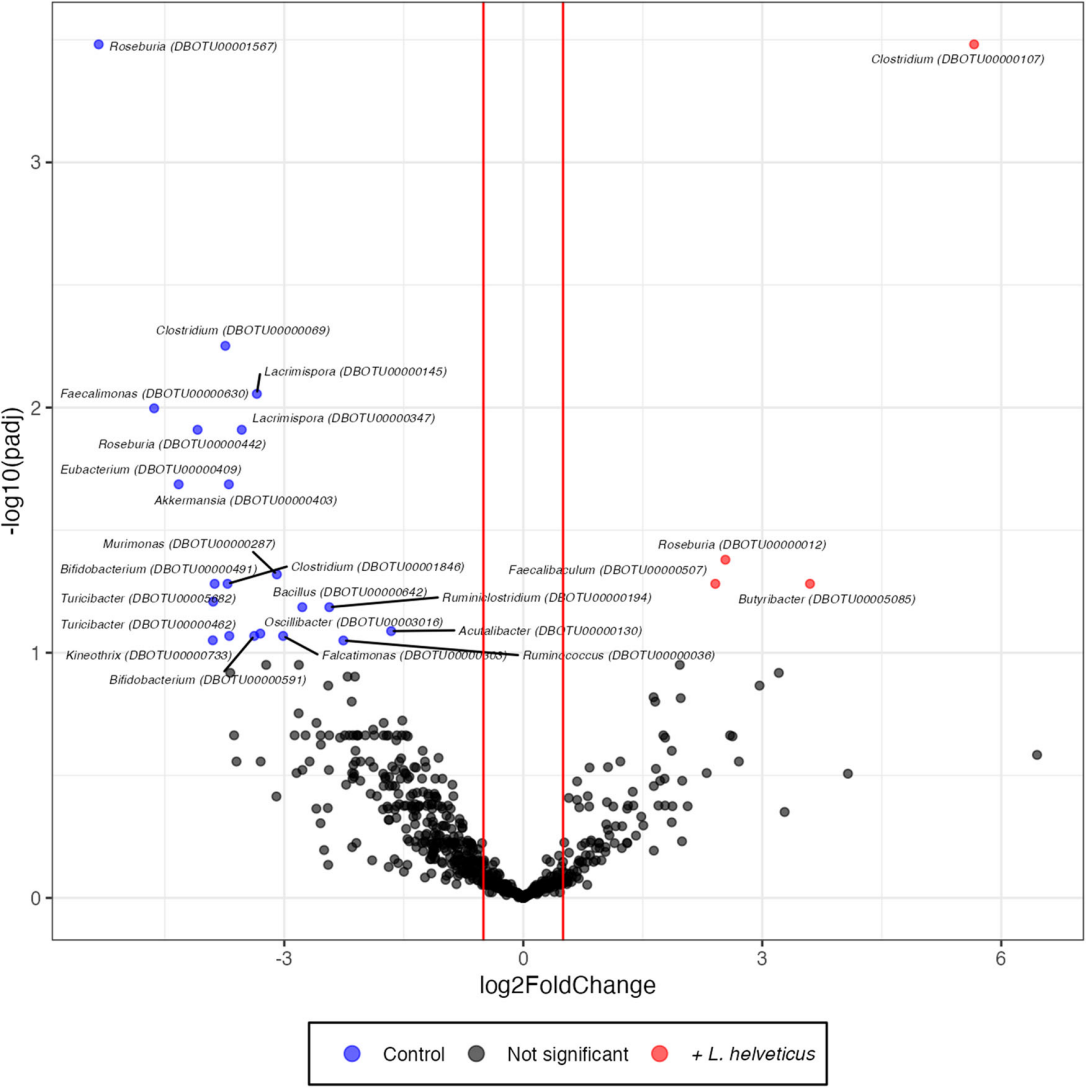
Volcano plot highlighting OTU fold changes in the gut microbiota of *L. helvet-icus*-supplemented mice. Each point represents an operational taxonomic unit (OTU). The x-axis represents the log2 of the fold change whilst the y-axis is the negative log10 of DESeq2 p values adjusted for multiple testing using the false discovery rate method. The vertical lines are fold-change cutoff that correspond to a log 2-fold change of 0.5 and 0.5. Blue points to the left of the plot with negative log2FoldChange values represent OTUs with increased abundance in control mice relative to mice fed *L. helveticus*-supplemented diet. Red points to the right of the plot with positive log2FoldChange values represent OTUs with increased abundance in mice fed *L. helveticus*-supplemented diet relative to control mice. *n* = 10/group.

To go further in the analysis of the microbial communities and find a pattern of bacterial species able to describe the changes in microbiota composition of *L. helveticus*-supplemented mice, we conducted a DESeq2 analysis at the OTU level. The DESeq2 differential abundance multiple-testing results were displayed on the volcano plot presented in Fig. 8. Microbiota analysis by 16S sequencing revealed no increase in the abundance of the *Lactobacillus* genus in the fecal microbiota of *L. helveticus-*supplemented mice compared to that of control mice (Fig. 8 and Supplementary Fig. 2). However, a 65-fold increase in the DNA amount of *L. helveticus* was shown by quantitative PCR in the feces of mice fed a *L. helveticus*-supplemented diet compared to those of mice fed a control diet confirming the enrichement in *L. helveticus* in supplemented mice (Supplementary Fig. 10b).

The abundance of 21 OTUs was significantly decreased (blue dots) and that of 4 OTUs (red dots) was significantly increased in the fecal microbiota of *L. helveticus*-supplemented mice compared to that of control mice (Fig. 8 and Supplementary Fig. 3). For each OTU identity, the seed sequence was selected and compared to the 16S ribosomal database of NCBI with BLASTN and default parameters (Table 1).

**Table 1:** Taxonomic identification of OTUs.

DBOTU	Phylum	Identification (BLASTN best match)	Identity (%)	Accession number	Log2FoldChange	Adjusted *p* value^1^
00000012	*Firmicutes*	*Roseburia faecis*	95.56	NR_042832.1	2.54	4.2E–02
00000036	*Firmicutes*	*[Ruminococcus] gnavus* ATCC 29149	93.32	NR_118690.1	-2.26	8.9E–02
00000069	*Firmicutes*	*[Clostridium] scindens*	98.27	NR_028785.1	-3.74	5.6E–03
00000107	*Firmicutes*	*Clostridium cellulovorans* 743B	88.45	NR_102875.1	5.66	3.3E–04
00000130	*Firmicutes*	*Acutalibacter muris*	100.00	NR_144605.1	-1.66	8.2E–02
00000145	*Firmicutes*	*Lacrimispora aerotolerans*	93.81	NR_119068.1	-3.34	8.8E–03
00000194	*Firmicutes*	*Ruminiclostridium cellulolyticum* H10	88.40	NG_041947.1	-2.43	6.5E–02
00000287	*Firmicutes*	*Murimonas intestini*	95.05	NR_134772.1	-3.09	4.8E–02
00000303	*Firmicutes*	*Falcatimonas natans*	92.57	NR_152688.1	-3.01	8.5E–02
00000347	*Firmicutes*	*Lacrimispora aerotolerans*	89.34	NR_119068.1	-3.53	1.2E–02
00000403	*Verrucomicrobia*	*Akkermansia muciniphila*	100.00	NR_074436.1	-3.70	2.1E–02
00000409	*Unknown*	*[Eubacterium] rectale* ATCC 33656	85.78	NR_074634.1	-4.33	2.1E–02
00000442	*Firmicutes*	*Roseburia faecis*	95.31	NR_042832.1	-4.09	1.2E–02
00000462	*Firmicutes*	*Turicibacter sanguinis*	88.55	NR_028816.1	-3.69	8.5E–02
00000491	*Firmicutes*	*Bifidobacterium animalis*	86.08	NR_043438.1	-3.87	5.2E–02
00000507	*Firmicutes*	*Faecalibaculum rodentium*	96.96	NR_146011.1	2.41	5.2E–02
00000591	*Actinobacteria*	*Bifidobacterium animalis*	83.92	NR_043438.1	-3.38	8.5E–02
00000630	*Actinobacteria*	*Faecalimonas umbilicata*	84.91	NR_156907.1	-4.63	1.0E–02
00000642	*Firmicutes*	*Bacillus anthracis*	95.77	NR_118536.1	-2.77	6.5E–02
00000733	*Firmicutes*	*Kineothrix alysoides*	95.05	NR_156081.1	-3.90	8.9E–02
00001567	*Firmicutes*	*Roseburia faecis*	95.06	NR_042832.1	-5.33	3.3E–04
00001846	*Firmicutes*	*[Clostridium] populeti*	95.56	NR_026103.1	-3.71	5.2E–02
00003016	*Firmicutes*	*Oscillibacter ruminantium GH1*	91.13	NR_118156.1	-3.30	8.4E–02
00005085	*Firmicutes*	*Butyribacter intestini*	95.80	NR_173596.1	3.60	5.2E–02
00005682	*Firmicutes*	*Turicibacter sanguinis*	93.47	NR_028816.1	-3.89	6.2E–02

^1^Adjusted *p*-value computed during the DeSeq2 analysis with the Benjamini and Hochberg method.

Among the 25 OTUs whose abundance were modified by the *L. helveticus* supplementation, 21 belonged to the *Firmicutes* phylum, 2 belonged to the *Actinobacteria* phylum, 1 belonged to the *Verrucomicrobia* phylum and 1 was not classified (Table 1). Twenty-three of the OTU sequences presented percentage identities of less than 98.5% when they were compared with the sequences of the NCBI database. However, among the *Firmicutes*-related OTUs, the sequence of OTU00000130 displayed 100% identity with the species *Acutalibacter muris*. In addition, the sequence of the *Verrucomicrobia*-related OTU (OTU00000403) matched with 100% identity to the species *Akkermansia muciniphila* (Table 1). No modification in the *Firmicutes*/*Bacteroidetes* ratio was observed (Supplementary Fig. 4).

### Correlation between retinal fatty acids and changes in the gut microbiota associated with long-term consumption of *L. helveticus*

**Fig. 9:**
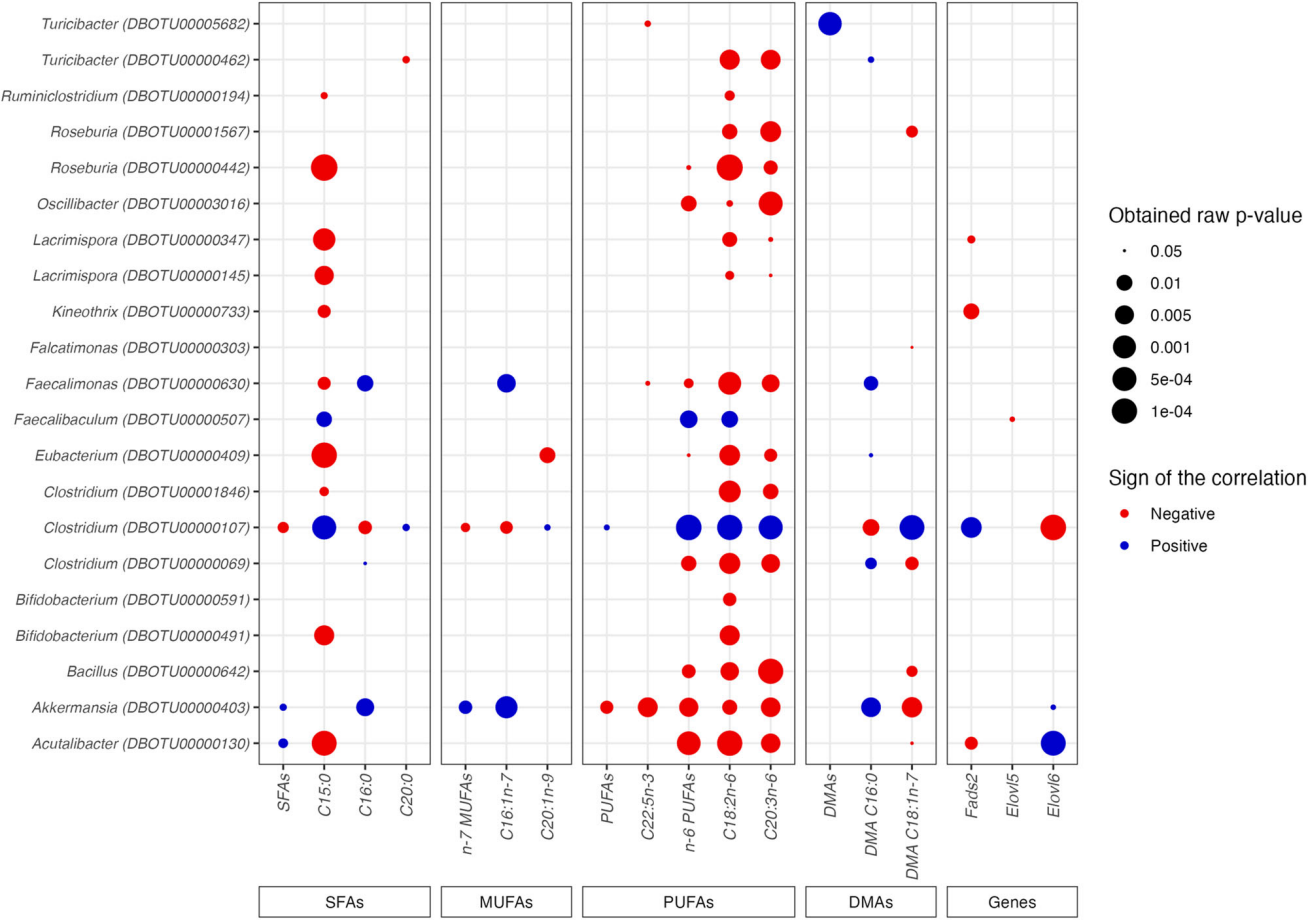
Dot plot of correlations obtained by applying a Spearman analysis to the normalized abundance of OTUs in the gut microbiota and the amount of fatty acids or the expression level of genes encoding enzymes involved in fatty acid biosynthesis in the retina. The normalized abundance of OTUs identified to be significantly different between the two groups of mice (control mice and mice fed a diet supplemented with *L. helveticus*) by DESeq2 were tested for correlation with the retinal amounts of fatty acids or the retinal expression levels of genes involved in fatty acid biosynthesis found to be significantly modified (or with a high tendency (*Elovl2 p* = *0.0524*)) between the two groups of mice by using Spearman linear correlations. Each correlation is represented by a circle whose size represents the obtained raw *p*-value, and whose color represents the correlation sign (blue corresponds to +1 r-values, indicating a positive linear correlation, and red corresponds to -1 r-values, indicating a negative linear correlation). *n* = 10/group.

To investigate any potential links between gut microbiota changes and the modifications of the FA content in the retina observed in *L. helveticus*-supplemented mice, we correlated the abundance of FAs or the expression level of enzymes involved in FA biosynthesis that were significantly modified in *L. helveticus*-supplemented mice with the normalized abundance of individual OTU identified by the DESeq2 analysis (Figs. 8, 9, and Supplementary Figs. 5–9). No correlation was found between the abundance of 4 OTUs (OTU00000012, OTU00000036, OTU00000287 and OTU00005085) and the retinal level of FAs or expression level of genes encoding FA-related enzymes. Regarding the other 21 OTUs, we observed that on the one hand the OTUs whose abundance was increased in the gut microbiota of *L. helveticus*-supplemented mice (OTU00000107 and OTU00000507) and on the other hand the OTUs whose abundance was decreased in the gut microbiota of *L. helveticus*-supplemented mice segregated (Fig. 9 and Supplementary Figs. 5–9). Indeed, when a positive correlation was found between the abundance of OTU00000107 and/or OTU00000507 and the retinal level of FAs or expression level of genes encoding FA-related enzymes, a negative correlation was found for the other OTUs, and conversely (Fig. 9 and Supplementary Figs. 5–9). Of note, we did not identify any positive and negative correlations between the OTUs significantly modified by the *L. helveticus* supplementation and the retinal amounts of total n-3 PUFAs, DHA (C22:6n-3) and C16:1n-9 (Fig. 9).

The abundances of two OTUs, OTU00000107 and OTU00000403, were oppositely correlated to 11 of the 18 changes observed in the retina at the FA or gene expression level. The abundance of OTU00000107 (*Firmicutes*, 88.45 % of identity with *Clostridium cellulovorans* 743B, Table 1) was positively correlated with the retinal amount of C15:0, C20:0, C20:1n-9, total PUFAs, n-6 PUFAs, C18:2n-6, C20:3n-6, DMA C18:1n-7 and *Fads2* expression in the retina, and negatively correlated with the retinal amount of total SFAs, C16:0, n-7 MUFAs, C16:1n-7, DMA C16:0 and *Elovl6* expression in the retina (Fig. 9 and Supplementary Figs. 5–9). The abundance of OTU00000403 (*Verrucomicrobia*, 100% identity with *A. muciniphila*, Table 1) was positively correlated with the retinal amount of total SFAs, C16:0, n-7 MUFAs, C16:1n-7 and DMA C16:0 and *Elovl6* expression in the retina, and negatively correlated with the retinal amount of total PUFAs, C22:5n-3, n-6 PUFAs, C18:2n-6, C20:3n-6 and DMA C18:1n-7 (Fig. 9 and Supplementary Figs. 5–9). In addition, we observed that a consortium of 9 OTUs was correlated (positive correlation: OTU00000107; negative correlation: OTU00000069, OTU00000130, OTU00000403, OTU00000409, OTU00000442, OTU00000630, OTU00000642 and OTU00003016) to the retinal amount of n-6 PUFAs (Fig. 9 and Supplementary Figs. 5–9). Finally, a negative correlation of the retinal amount of C22:5n-3 (docosapentaenoic acid, DPA) and the abundance of a consortium of 3 OTUs (OTU00000403, OTU00000630 and OTU00005682) was observed.

## Discussion

PUFAs of the n-3 series and their derivatives are crucial for the retinal physiology and play a pivotal protective role in AMD^8^. In light of recent literature supporting the existence of a gut microbiota-retina axis and studies showing the influence of the gut microbiota on host FA metabolism, targeting the gut microbiota to modulate the FA content of the retina seems an attractive strategy to prevent retinopathies such as AMD. The aim of this study was to investigate in mice the effect of long-term consumption of *L. helveticus* strain VEL12193 on the composition of the gut microbiota, the host lipid metabolism and the FA content of the retina.

Evidence has accumulated showing the influence of the gut microbiota on the host lipid metabolism through the modulation of key metabolic pathways in the liver, including those involved in cholesterol metabolism^35^. A cholesterol-lowering effect has been described for several species of lactobacilli^36–39^. In this study, we showed that long-term consumption of *L. helveticus* VEL12193 did not modify the cholesterol level in the liver and the plasma. It should be noted that few studies have investigated the effect of *L. helveticus* species on cholesterol metabolism and their results are not consensual^40,41^.

The liver is a key organ in lipid metabolism. The analysis of its FA content revealed that long-term supplementation with *L. helveticus* affected the amount of total SFAs. This phenotype could be directly related to the hepatic decrease observed in the major FA of this class, namely palmitic acid (C16:0). It is unlikely that the alteration of the C16:0 level was due to a difference in the diet composition since the incorporation of the bacteria into the dietary preparation did not modify the amounts of FA. In addition, no modulation of the hepatic expression level of genes encoding enzymes involved in FA biosynthesis was observed including for *Fasn* that encodes FAS, an enzyme involved in de novo lipogenesis and whose primary reaction product is C16:0. Another hypothesis that could explain the decrease in the hepatic C16:0 amount is an alteration in its intestinal absorption and/or in its esterification/transfer into lipoproteins. To test this hypothesis, the intestinal expression level of genes encoding acylglycerol transferase (MOGAT, DGAT) and proteins involved in FA uptake (e.g., CD36) and lipoprotein assembly (e.g., MTTP) could be analyzed in *L. helveticus*-supplemented mice. Finally, it has been reported that *Lacticaseibacillus rhamnosus* strain GG has the ability to consume FAs, including C16:0, a property that reduces intestinal FA absorption^42^.

Plasma FA content was more importantly affected by the *L. helveticus* supplementation than liver since changes were observed among SFAs (decrease in C14:0, C16:0 and total SFA amounts; increase in C20:0 and C22:0 amounts), MUFAs (decrease in C16:1n-9 amount; increase in C22:1n-9 amount) and also PUFAs (increase in total PUFAs, total n-6 and n-3 PUFAs, and C20:2n-6). The DMA C16:0 amount was also altered. The *L. helveticus*-associated plasma alterations in C16:0 and total SFAs may directly reflect the FA liver status of these FAs. It is likely that this is also the case for the decrease in DMA C16:0, since no difference in the expression of genes encoding plasmalogen synthesis enzymes (*Far1*, *Agps*, and *Gnpat*) was observed in *L. helveticus*-supplemented mice. We can also assume that the decrease in C16:1n-9 may result from reduced hepatic C16:0 abundance rather than a desaturation defect since the hepatic expression of the gene encoding SCD-1, which catalyzes insertion of the double bond at the delta-9 position of C16:0, was unchanged in *L. helveticus* supplemented mice. As a consequence of the plasma decreases in SFAs and MUFAs, the relative abundance of PUFAs was increased. However, neither the liver PUFA content nor the hepatic expression of genes encoding elongase and desaturase were modified in *L. helveticus*-supplemented mice.

As expected, some of the changes observed in the plasma of *L. helveticus*-supplemented mice were also found in the retina, particularly those affecting C16:0 and its MUFA derivatives. These changes were balanced by an increase in the abundance of PUFAs. At the species level, retinal alterations in PUFAs were different from those observed in the plasma where only an increase in the level of C20:2n-6 and an upward trend for that of C22:6n-3 were measured. Among the n-6 PUFAs, we noticed an increased retinal incorporation of C18:2n-6 (linoleic acid, LA), which is the precursor for n-6 PUFAs that is only provided by the diet. This phenotype could result from an enhanced expression of FA transporters at the retinal barrier such as FATPs (fatty acid transport proteins), FABPs (fatty acid binding proteins) or FA translocase^43^. In an in vitro model of human brain microvascular endothelial cells, it has been shown that FATP4-knockdown reduced the transport of LA, thus indicating that this protein is involved in LA transport^44^. Interestingly, some gut microbes could influence the expression level of these proteins in organs/tissues. Indeed, treatment of mice with *A. muciniphila* activated hepatic expression of *Fatp4* and *Cd36*^45^. However, in our study supplementing mice with *L. helveticus* VEL12193 was associated with a reduction of the OTU403, assigned to *A. muciniphila*, in the fecal microbiota.

In addition to LA, the abundance of C20:3n-6 (dihomo-gamma-linolenic acid, DGLA) was increased in the retina of *L. helveticus*-supplemented mice. DGLA is the elongation product of C18:3n-6 (gamma-linolenic acid, GLA) that it is itself the desaturation product of LA. Once produced, DGLA can integrate two different pathways that can lead to the production of bioactive molecules (eicosanoids) with different inflammatory properties. On the one hand DGLA can serve as a precursor for the biosynthesis of prostaglandins and thromboxane of the series-1 that are generally viewed as having mainly anti-inflammatory properties. But, on the other hand, desaturation of DGLA by FADS2 will lead to the production of C20:4n-6 (arachidonic acid, AA), a precursor of prostaglandins and thromboxane of the series-2 and leukotrienes of the series-4, having mainly pro-inflammatory properties. No modification in the amount of C20:4n-6 was observed in the retina of *L. helveticus* supplemented mice suggesting that DGLA is probably not desaturated. However, further experiments that could give information on the retina inflammatory status are needed to conclude on the beneficial *versus* harmful effect of increased DGLA level observed in the retina of *L. helveticus* supplemented-mice.

Several studies support that a diet enriched in n-3 LC-PUFAs as well as high concentration of plasma n-3 PUFAs are protective against AMD^8,46–48^. Conversely, low dietary intake of n-3 LC-PUFAs has been correlated with a higher risk of developing the disease^8,47^. Interestingly, we showed that dietary supplementation with *L. helveticus* VEL12193 was associated with an increase in retinal n-3 PUFAs. More specifically, the amounts of two n-3 PUFA were increased in the retina of *L. helveticus*-supplemented mice compared to controls: C22:5n-3 (n-3 docosapentaenoic acid, n-3 DPA) and DHA. DPA is an intermediate product between C20:5n-3 (eicosapentanoic acid, EPA) and DHA. In vitro and in vivo studies have shown that DPA can be retro-converted into EPA^49^. Retinal DPA conversion to DHA has also been reported in miniature poodle dogs^50^. Many beneficial biological effects have been described for EPA and DHA in the retina, including protection against oxidative stress, neovascularization and inflammation, which are mechanisms involved in AMD^51^. In addition, although its biological effects have been until now under-explored, DPA may also possess beneficial properties for retinal health^49^.

Predominant lipids in the retina are phospholipids, with phosphatidylcholine (PCs) and phosphatidylethanolamine (PEs) accounting for the majority (≈ 90%) of the retinal phospholipids^1,2^. In accordance with previous studies, we observed that the two predominant retinal PCs species contain disaturated FAs (PC(16:0/16:0), 17.5% of total PCs) and saturated/monounsaturated FAs (PC(16:0/18:1), 17.9% of total PCs), whereas the two major PEs species contain saturated/polyunsaturated (DHA) FAs (PE(18:0/22:6), 26.1% of total PEs; PE(16:0/22:6), 14.8% of total PEs). Interestingly, long-term dietary supplementation with *L. helveticus* enriched the retinal content of two PE species esterified with DHA, including PE(16:0/22:6).

Spearman’s correlative analyses between OTUs whose abundance was altered by *L. helveticus* supplementation and retinal FAs abundance have allowed us to identify some potential correlations. However, a limitation of our study is the small size of the sample. Only raw P-values are presented without multiple hypothesis correction, a condition which raises the possibility of false positive results. Regarding the obtained correlations, a consortium of 9 OTUs associated with retinal n-6 PUFA changes and a consortium of 3 OTUs associated with retinal n-3 PUFA changes have been identified. Two OTUs were common to these 2 consortia: OTU630, belonging to Actinobacteria and whose sequence has 84.91% of identity with *Faecalimonas umbilicata* and OTU403, belonging to *Verrucomicrobia* and whose sequence has 100% identity with *A. muciniphila*^52^. Such an observation raises the possibility that changes in the FA content of the retina associated with *L. helveticus* supplementation resulted from a reshaping of the gut microbiota composition rather than the action of a unique bacteria strain. Indeed, reshaping of the gut microbiota can affect its metabolic functions and thus modify its communication with the host at the level of the gut mucosa but also at the level of other distant organs. Some studies suggest that some products derived from the metabolic activities of the gut microbiota such as SCFAs (e.g., propionate, butyrate) and secondary bile acids (e.g., ursodeoxycholic acid, UDCA; tauroursodeoxycholic acid, TUDCA) could take part in the dialogue between the gut microbiota and the eye^53–61^. Interestingly, in addition to SCFAs, studies suggest that UDCA and TUDCA could also be involved in the regulation of the host FA metabolism^17,62,63^. To further understand the molecular mechanisms linking the reshaping of the gut microbiota induced by *L. helveticus* and its consequences on the bioavailability of FAs for the retina, an analysis of the gut microbiota-derived metabolites at the gut and systemic levels is required.

Finally, we cannot exclude that lipid changes observed in *L. helveticus* supplemented mice could be driven, at least in part, by changes in energy metabolism or in lean and fat body masses. Previous studies from our group have shown that consumption of high fructose diet impacted rat fat mass without affecting their body weight. This phenotype was associated with the modulation of the hepatic lipid and fatty acid composition, as well as changes in the expression of genes involved in lipid metabolism in the retina^64,65^. In this study we showed that long-term consumption of *L. helveticus* strain VEL12193 limited weight gain in mice. However, other studies have suggested that lactobacilli could exert different effects on body weight depending on several factors that include the bacterial species or strains studied, the study model, the mouse diet, and the mode of administration of the bacteria^66–68^. In our conditions, the limitation in weight gain of *L. helveticus*-supplemented mice was neither associated with a reduction in visceral fat nor a lower food intake. However, further investigations are needed to more accurately characterize changes in energy metabolism and/or in lean and fat masses induced by *L. helveticus*. Several hypotheses could be explored to characterize the origin of this *L. helveticus* VEL12193-related phenotype. The first-one would be a loss of muscle and/or bone mass, but such a hypothesis is unlikely since lactobacilli have been shown to instead have the opposite effect^69–72^.

This hypothesis is also supported by studies in animal models showing that gut microbiota influences host energy metabolism^73^. *L. helveticus* VEL12193 could have modified of the gut microbiota capacities on nutrient absorption, energy expenditure and/or fat oxidation^74,75^. Although results in humans are inconsistent, an increased *Firmicutes*/*Bacteroidetes* ratio has been reported to modify the metabolic function of the gut microbiota, including the production of short chain fatty acids (SCFAs; e.g., acetate, propionate, and butyrate) that could be involved in body weight control^76–79^. No modification of the *Firmicutes*/*Bacteroidetes* ratio was observed in *L. helveticus*-supplemented mice. However, the sequences of the 4 OTUs whose abundances were increased in the gut microbiota by *L. helveticus* supplementation were assigned to 4 bacterial genera encompassing some species that are known as butyrate-producers (namely *Roseburia faecis*, *Clostridium cellulovorans*, *Faecalibaculum rodentium* and *Butyribacter intestini*)^80–83^. Whether *L. helveticus* VEL12193-supplementation impacts SCFAs production by the gut microbiota remains to be determined.

In conclusion, we showed that long-term dietary supplementation with *L. helveticus* enriched the retina in DGLA and DHA that are two PUFAs having beneficial health properties that could help to protect the retina against deleterious age-related mechanisms/stresses. These *L. helveticus*-induced retinal lipid modifications were associated with a reshaping of the gut microbiota composition. Further investigations are now required to (i) determine whether the PUFA-enrichment induced by long-term consumption of *L. helveticus* would be effective in protecting the retina from the harmful effects of aging and (ii) identify the molecular actors linking the changes induced by *L. helveticus* in the gut microbiota and their effect on the retinal physiology.

## Methods

### Mice

The use of animals was in accordance with the ARVO Statement for the Use of Animals in Ophthalmic and Vision Research. French legal and institutional ethics committee review board approvals were obtained (2018072513005644).

Eight-week-old male C57BL/6JRj SPF mice were purchased from Janvier Labs, France. They were maintained at INRAE, Dijon, France until euthanasia (C21 231 010 EA) with *ad libitum* access to food and water and exposed to 12 h:12 h light:dark cycles. After one week of acclimation, mice were randomly divided into two groups: one group received standard diet (control group; *n* = 10) and the other group received the same diet as controls but supplemented with *L. helveticus* (*L. helveticus* group; *n* = 10). Mice were maintained on these diets for 6 months. Fecal samples were collected for microbiota analyses one day before the end of the experiment. Prior to euthanasia, mice were fasted for 15 h in order to avoid any interference of the food intake with blood lipid analyzes. They were euthanized by cervical dislocation. Liver, retina and blood were collected. Hemolysis-free serum was generated by centrifugation (1800× *g*, 10 min, 4 °C).

### Diet

*L. helveticus* strain VEL12193 has been isolated from cheese^33^. It was grown overnight under anaerobic conditions at 37 °C without shaking in Man-Rogosa-Sharpe medium (Condalab), pH 5.8. The bacterial culture was centrifuged at 5000 *g* for 10 min at room temperature. The bacterial pellet was washed twice in PBS and resuspended in sterile water at a concentration of 2.10^9^ CFUs/mL. This bacterial suspension was then mixed with complete maintenance diet powder for adult mice (SAFE® A04) to obtain a final bacterial concentration in the diet of 1.10^9^ CFUs/g. Food portions (approximatively 20 g) were molded into Petri dishes, dried for 24 h at 4 °C and then stored anaerobically at 4 °C. Fresh diet was prepared weekly. The food portion were renewed in the cages every 2 days from the stock stored in anaerobic conditions at 4 °C. The viability of *L. helveticus* in food portions stored under these conditions was checked (Supplementary Fig. 10). The FA content of the diets is provided in Supplementary Table 5.

### Quantification of *L. helveticus* DNA by qPCR

Fresh feces were collected and total DNA was extracted and purified using ZymoBIOMICS DNA Miniprep kit (Zymo Research). The quantity of *L. helveticus* DNA was determined by quantitative real-time PCR analysis using iTaq universal SYBR Green supermix (Bio-Rad). Primers targeting the phenylalanyl-tRNA synthase gene (*pheS*) of *L. helveticus* were used: “*L. helveticus* forward” 5’-AGGTTCAAAGCATCCAATCAATATT-3’ and “*L. helveticus* reverse” 5’-TCGGGACCTTGCACTACTTTATAAC-3’^84^. The quantity of total eubacteria was determined by qPCR using primers targeting the 16S rRNA gene: “Total eubacteria forward” 5’-CAGCAGCCGCGGTAATAC-3’ and “Total eubacteria reverse” 5’-CCGTCAATTCCTTTGAGTTT-3’. Quantitative real-time PCR was performed on the CFX96 PCR system (Bio-Rad). Fold changes in *L. helveticus* DNA were calculated using the ΔΔCt method of relative quantification, with the total bacteria as a reference.

### Microbiota analysis

One day before euthanasia and before starving, fresh feces (3-4 pellets/mouse) were collected into sterile tubes, snap-frozen dry in liquid nitrogen and stored at −80 °C. Collection of the feces was performed on the same time slot for all mice. An optimized and standardized DNA extraction protocol dedicated to bacterial DNA extraction from stool samples has been used (GenoScreen, Lille, France).

DNA has been extracted from the samples (≈ 200 mg of feces) using an optimized protocol partially based on commercially available extraction kits (QIAamp® DNA stool Kit, Qiagen) including GenoScreen in-house lysis steps combining mechanical and chemical methods. At this step, negative controls BG-Ext (Extraction protocol applied on sterile water) have been regularly introduced. These BG-Ext are also called Kitome. After DNA extraction, the concentration was determined with the SybrGreen assay Kit (Life Technologies, USA).

A 16S rRNA gene fragment comprising V3 and V4 hypervariable regions was amplified using an optimized and standardized 16S-amplicon-library preparation protocol (Metabiote®, GenoScreen, Lille, France). Briefly, 16S rRNA gene PCR was carried out using 5 ng of genomic DNA according to Metabiote® protocol (or maximal of DNA volume) instructions using 192 bar-coded primers (Metabiote® MiSeq Primers, GenoScreen, Lille, France) at final concentrations of 0.2 μM and an annealing temperature of 50 °C for 30 cycles. PCR products were cleaned up with Agencourt AMPure XP-PCR Purification system (Beckman Coulter, Brea, USA), quantified according to the manufacturer’s protocol, and multiplexed at equal concentration. Sequencing was performed using a 250-bp paired-end sequencing protocol on the Illumina MiSeq platform (Illumina, San Diego, USA) at GenoScreen, Lille, France.

Bioinformatic analyses were performed using the BIOCOM-PIPE pipeline, with default parameters, except when parameters were clearly described^85^. First, the 16S raw reads were sorted according to each sample using multiplex identifiers, and low-quality reads were deleted based on their length (less than 350-bp for 16S reads), their number of ambiguities and their primer(s) sequence(s). Then a PERL program was applied for rigorous dereplication (i.e. clustering of strictly identical sequences). The dereplicated reads were globally aligned using the Infernal tool^86^, and clustered into operational taxonomic units (OTUs) using a similarity threshold of 97%. A filtering step was carried out to remove chimeras based on the quality of their taxonomic assignments. Finally, the retained reads were homogenized by random selection (28,663 reads for 16S rRNA gene sequences) to compare the datasets efficiently and avoid biased community comparisons. The retained high-quality reads were used to determine alpha-diversity metrics after clustering refining with ReClustOR to improve OTUs definition^87^, and taxonomy-based analysis was performed using USEARCH against the SILVA 16 S rRNA reference database (r132).

The raw datasets are available in the EBI database system under project accession number PRJEB56822.

### Lipid class distributions

Total lipids were extracted from plasma, livers and retinas following the Folch’s procedure^88^. The distribution of lipids into different classes [phospholipids (PL), triglycerides (TG), diglycerides (DG), free fatty acids (FFA), free cholesterol (Chol), and/or cholesteryl esters (CE)] was determined using a combination of thin-layer chromatography on silica gel-coated quartz rods and flame ionization detection (Iatroscan® system, Iatron, Tokyo, Japan), according to Ackman’s technique^89^. The values obtained for each compound were corrected according to their response factor using specific calibration curves. Data were reported as a percentage relative to total lipids in the sample (considered as 100%).

### FAME and DMA profiles

Total lipids were extracted as described above^88^. Boron trifluoride in methanol was used for transmethylation^90^. Hexane was used to extract fatty methyl esters (FAMEs) and dimethyl acetals (DMAs). Analyses were performed on a GC Trace 1310 (Thermo Scientific) gas chromatograph (GC) using a CPSIL-88 column (100 × 0.25 mm i.d., film thickness 0.20 μm; Varian). This device was coupled to a flame ionization detector (FID). The configuration was: inlet pressure of hydrogen 210 kPa, oven temperature 60 °C for 5 min + 165 °C at 15 °C per min and upholding for 1 min, + 225 °C at 2 °C per min and upholding at 225 °C for 17 min. The injector and the detector were maintained at 250 °C. Comparisons with commercial and synthetic standards enabled the identification of FAMEs and DMAs. The ChromQuest software (Thermo Scientific) was used to process the data.

### Analysis of phospholipid molecular species by liquid chromatography coupled to high-resolution mass spectrometer

Phosphorus content of the total lipid extract was determined according to the method developed by Bartlett and Lewis^91^. The total phospholipids were dried under a stream of nitrogen and diluted to the appropriate concentration of 500 µg/µL of phospholipids in CHCl3/CH3OH (1:1, v/v). Ten microliters of internal standard mixture containing PC(14:0/14:0) 320 µg/mL, PE(14:0/14:0) 160 µg/mL, PS(14:0/14:0) 80 µg/mL, PI(8:0/8:0) 100 µg/mL, and SM(d18:1/12:0) 80 µg/mL were added into 200 µL of this phospholipid solution.

Phospholipid classes were separated under hydrophilic interaction liquid chromatograph (HILIC) conditions using a Kinetex HILIC 100 × 2.1-mm, 1.7-µm column (Phenomenex, Sydney, NSW, Australia) as described previously^92^. Ultra-high-performance liquid chomatography (UHPLC) separation was achieved using an ULTIMATE 3000 LC pump and an ULTIMATE 3000 Autosampler (Thermo Scientific, San Jose, CA, USA). The mobile phase consisted of (A) CH3CN/H2O (96/4, v/v) containing 10 mM ammonium acetate and (B) CH3CN/H2O (50/50, v/v) containing 10 mM ammonium acetate. The chosen solvent-gradient system of the analytical pump was as follows : 0 min 100% A, 12 min 80% A, 18 min 50% A, 18.1–30 min 100% A. The flow rate was 500 μL/min, the injection volume was 10 µL and the column was maintained at 50 °C. The liquid chromatography system was controlled by Standard Instrument Integration (SII) software based on Dionex Chromeleon TN 7.

The process of identification and quantification of phospholipid species was performed on an orbitrap FusionTM Tribrid Mass Spectrometer equipped with an EASY-MAX NG Ion Source (Heated Electrospray Ionization H-ESI) (Thermo Scientific, San Jose, CA, USA). Phospholipid species were detected by high-resolution mass spectrometry (HRMS) analysis. H-ESI source parameters were optimized and set as follows: ion transfer tube temperature of 285 °C, vaporizer temperature of 370 °C, sheath gas flow rate of 35 au, sweep gas of 1 au, auxiliary gas flow rate of 25 au. Positive and negative ions were monitored alternatively by switching the polarity approach with a static spray voltage at 3500 V and 2800 V in positive and negative mode respectively. Mass spectra in full scan mode were obtained using the Orbitrap mass analyzer with the normal mass range and a target resolution of 240,000 (FWHM at m/z 200), in a mass-to-charge ratio m/z ranging from 200 to 1600 using a Quadrupole isolation in a normal mass range. All MS data were recorded using a maximum injection time of 100 ms, automatic gain control (AGC) target (%) at 112.5, RF lens (%) at 50, and one microscan. An intensity threshold filter of 1.103 counts was applied.

For tandem mass spectrometry (MS/MS) analyses, the data-dependent mode was used for the characterization of phospholipid species. Precursor isolation was performed in the Quadrupole analyzer with an isolation width of m/z 1.6. Higher-energy collisional dissociation was employed for the fragmentation of phospholipid species with an optimized stepped collision energy of 27%. The linear ion trap was used to acquire spectra for fragment ions in data-dependent mode. The AGC target was set to 2.104 with a maximum injection time of 50 ms. All MS and MS/MS data were acquired in the profile mode. The Orbitrap Fusion was controlled by XcaliburTM 4.1 software (Thermo Scientific, San Jose, CA, USA). The identification of all PL species was performed using the high-accuracy data and the information collected from fragmentation spectra with the help of LIPIDSEARCH software version 4.1.16 (Thermo Scientific, San Jose, CA, USA) and the LIPID MAPS® database (https://www.lipidmaps.org/).

### Gene expression

Total RNA was extracted using TRIzol reagent (Life Technologies). Reverse transcription was performed using PrimeScript RT reagent Kit with gDNA Eraser (Takara Bio). Gene expression was determined by real-time PCR using SYBR Green (Biorad) and a CFX96 Real-Time PCR system (Biorad). Hprt was used as the internal control for normalization. Primer sequences are given in Supplementary Table 6.

### Statistical analyses

The data are presented as mean ± standard deviation of the mean (s.e.m.), except those including bacterial communities. Statistical analyses were performed using the GraphPad Prism software for all analyses except those including bacterial communities, which were performed with R (version 4.1.2). The non-parametric Mann and Whitney or Kruskal-Wallis tests were used to compare data from the two groups (after Bonferroni correction). The p-values of less than 0.05 were considered statistically significant (*p < 0.05, **p < 0.01, ***p < 0.001 and ****p < 0.0001). Measurements were taken from distinct samples.

The cage effect was checked prior analysis, regarding both taxonomically dependent (at *phylum*, class and *genus* levels) and independent analyses (at the OTU level). A first filter was applied to reduce the OTU dataset, keeping only OTUs with at least 100 high-quality reads. Then, for each dataset (centered but not scaled), a Bray-Curtis distance matrix was computed with the ‘vegdist’ function from the vegan package. Then, a PERmutational Multivariate ANalysis of VAriance (PERMANOVA) analysis was realized to check for differences between cages (number of permutations: 1000, *p-value* < 0.05) using the ‘adonis2’ function from the adonis package. No cage effect was highlighted for the analyzed datasets.

OTUs differences between the two groups (mice receiving *L. helveticus* against those of the control group) were assessed by pairwise comparison of normalized sequence counts using Negative Binomial Wald Tests from the DESeq2 package^93^. More precisely, the DESeq2 package performed three steps: (1) estimation of size factors, which are used to normalize library sizes in a model-based fashion; (2) estimation of dispersions from the negative binomial likelihood for each feature, and subsequent shrinkage of each dispersion estimate towards the local trendline by empirical Bayes; (3) fitting each feature to the specified class groupings with negative binomial generalized linear models and performing hypothesis testing, for which we chose the default Wald test. Then, DeSeq2 helped to decrease the false discovery rate of OTUs, using the Benjamini and Hochberg method by default. The adjusted *p*-values < 0.1 were considered as significant for the DeSeq2 analysis.

To compare the OTUs modified by the probiotic supplementation with either fatty acid amounts or gene expression levels, Spearman correlation analyses (and related raw *p*-values) were performed with the ‘cor.test’ function from R, considering a *p*-value less than 0.05 as significant, using normalized OTUs abundances obtained after DeSeq2 analysis.

### Reporting summary

Further information on research design is available in the Nature Research Reporting Summary linked to this article.

### Supplementary information

Supplementary information

Reporting Summary

## Data Availability

For microbiota, the raw datasets are available in the EBI database system under project accession number PRJEB56822. All other data supporting the findings reported herein are available on reasonable request from the corresponding author.
