# Supplementary material for: Long-term intake of Lactobacillus helveticus enhances bioavailability of omega-3 fatty acids in the mouse retina
Source: NPJ Biofilms Microbiomes. 2024 Jan 18;10:4. doi: 10.1038/s41522-023-00474-5 (PMC10796366; doi:10.1038/s41522-023-00474-5)
Supplement: Supplementary file 1 — Supplementary information [file 41522_2023_474_MOESM1_ESM.pdf]

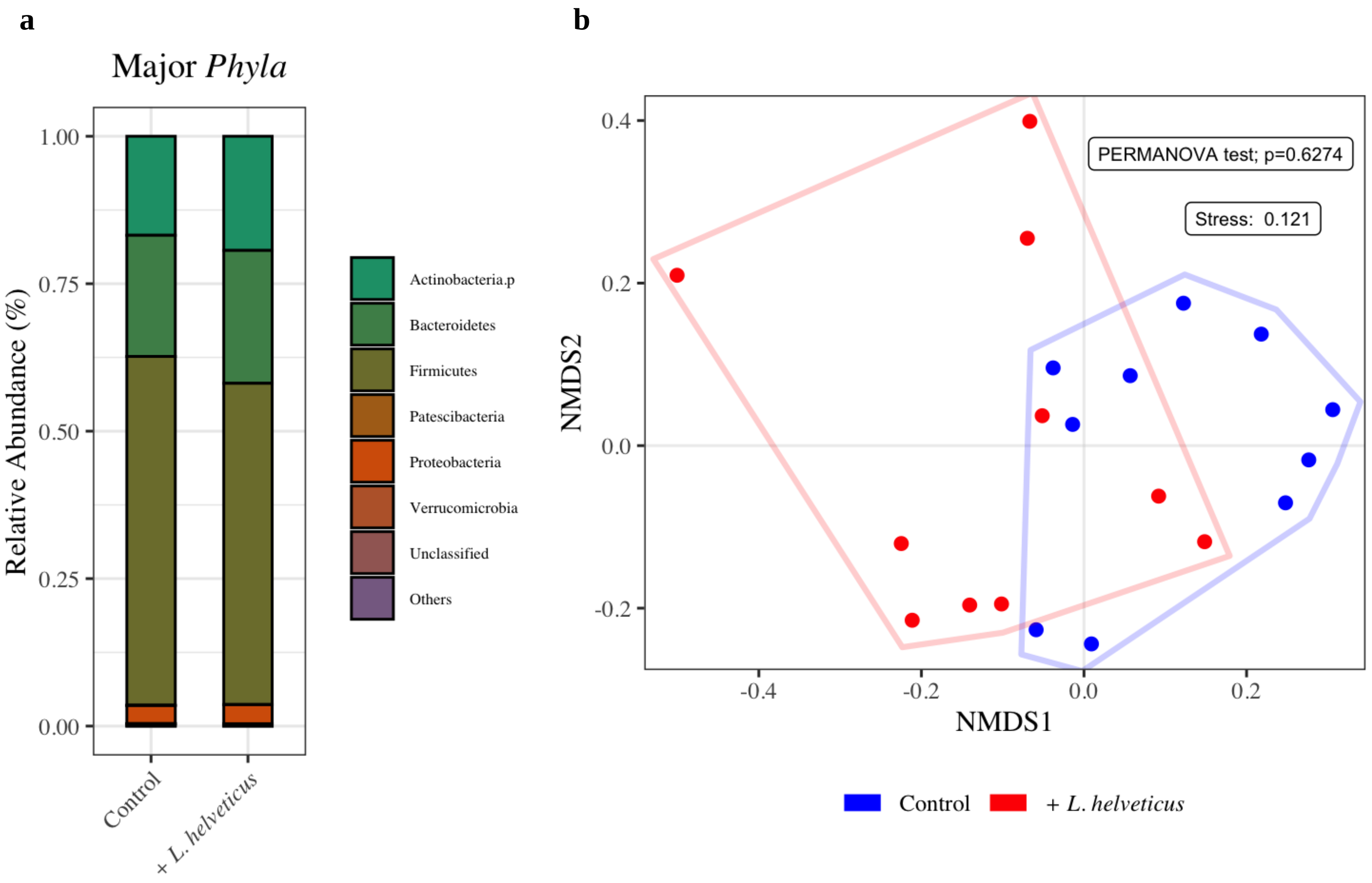

Supplementary Figure 1

Supplementary Figure 1. Relative abundances of the major phyla in the gut microbiota of *L. helveticus*-supplemented mice. (**a**) bar graph presenting the relative abundance of the major phyla in the gut microbiota of control mice and in that of mice fed a diet supplemented with *L. helveticus*. (**b**) Non-metric multidimensional scaling (NMDS) ordination of communities at the phylum level. n=10/group.

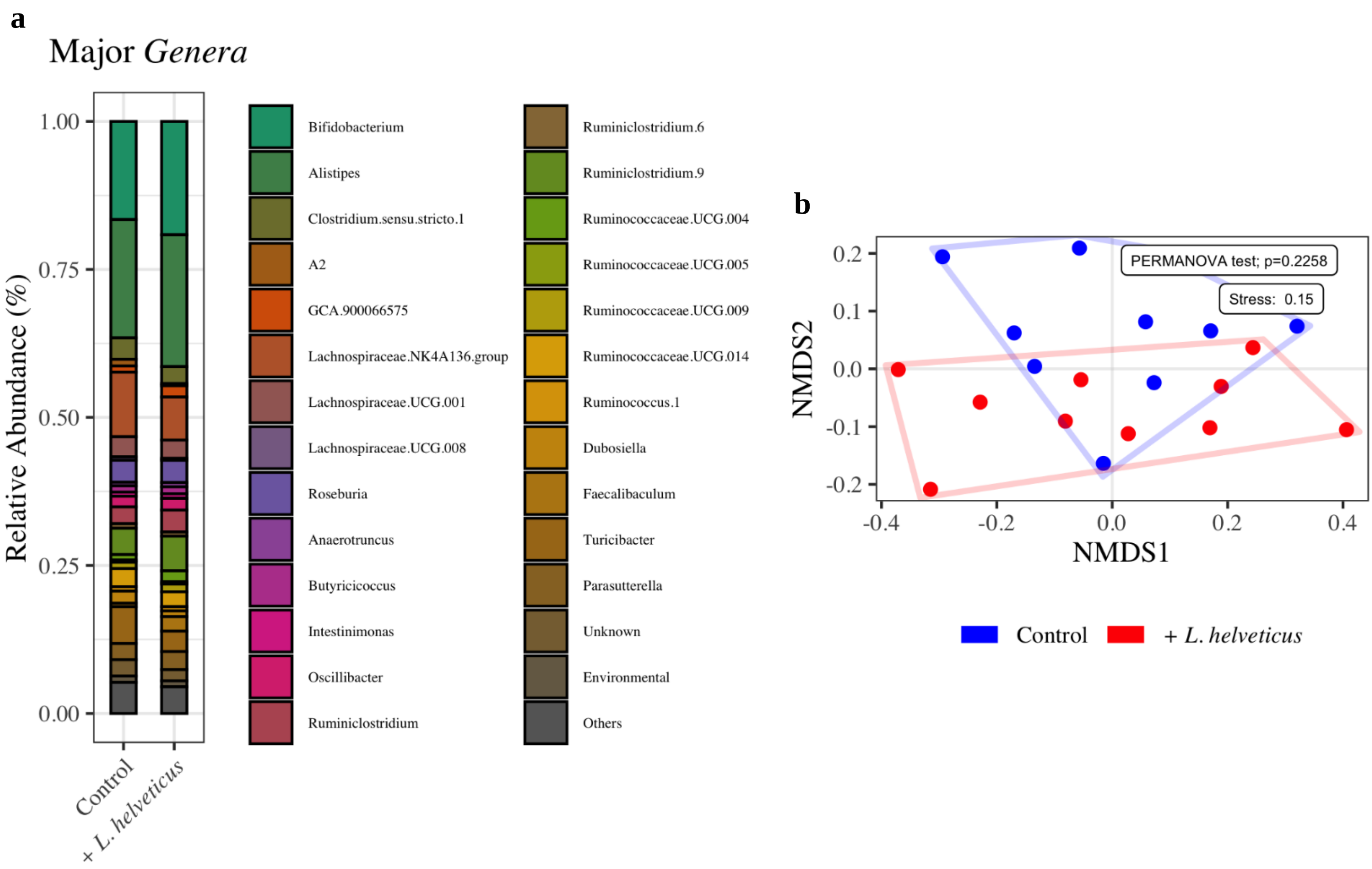

Supplementary Figure 2

Supplementary Figure 2. Relative abundances of the major genera in the gut microbiota of *L. helveticus*-supplemented mice. (**a**) bar graph presenting the relative abundance of the major genera in the gut microbiota of control mice and in that of mice fed a diet supplemented with *L. helveticus*. (**b**) Non-metric multidimensional scaling (NMDS) ordination of communities at the genus level. n=10/group.

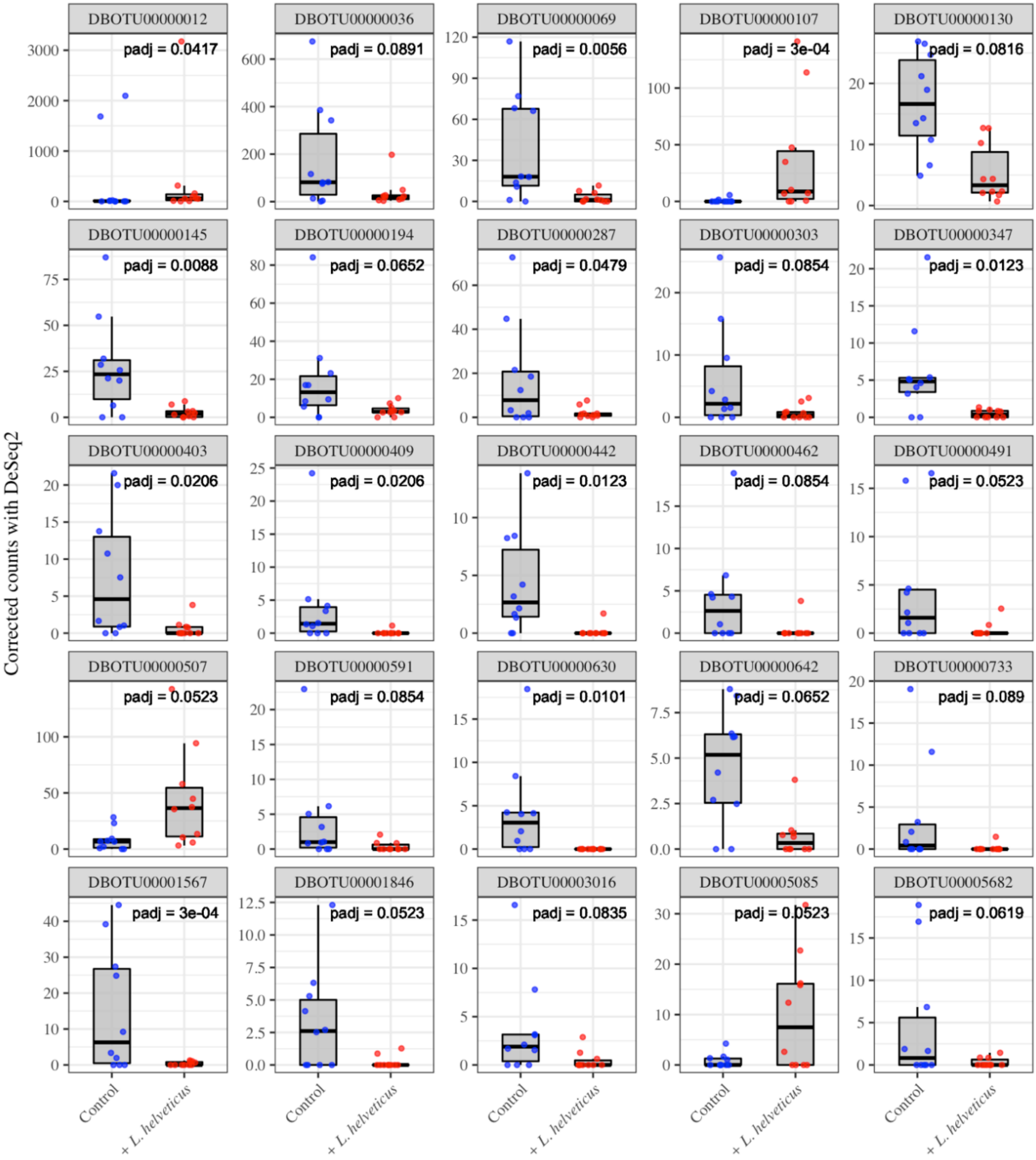

Supplementary Figure 3

Supplementary Figure 3. Relative abundances of OTUs identified by DESeq2 differential abundance multiple-testing. Data are presented as as corrected values for each OTU after DESeq2 normalization, with adjusted p-values (padj). Data are presented in box plot format (median and quartiles). n=10/group.

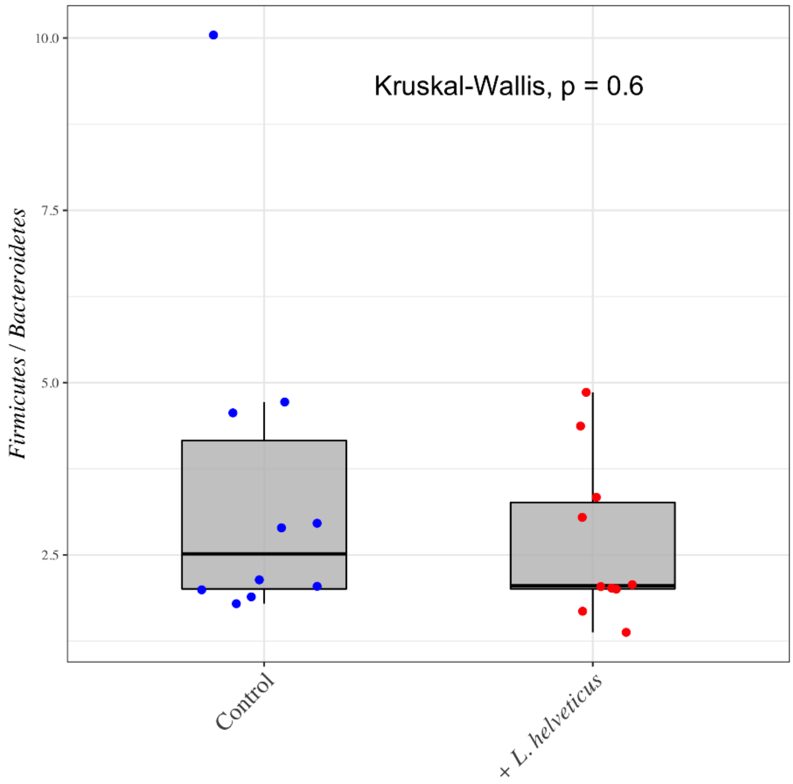

Supplementary Figure 4

Supplementary Figure 4. *Firmicutes*/*Bacteroidetes* ratio. Data are presented in box plot format (median and quartiles). n=10/group.

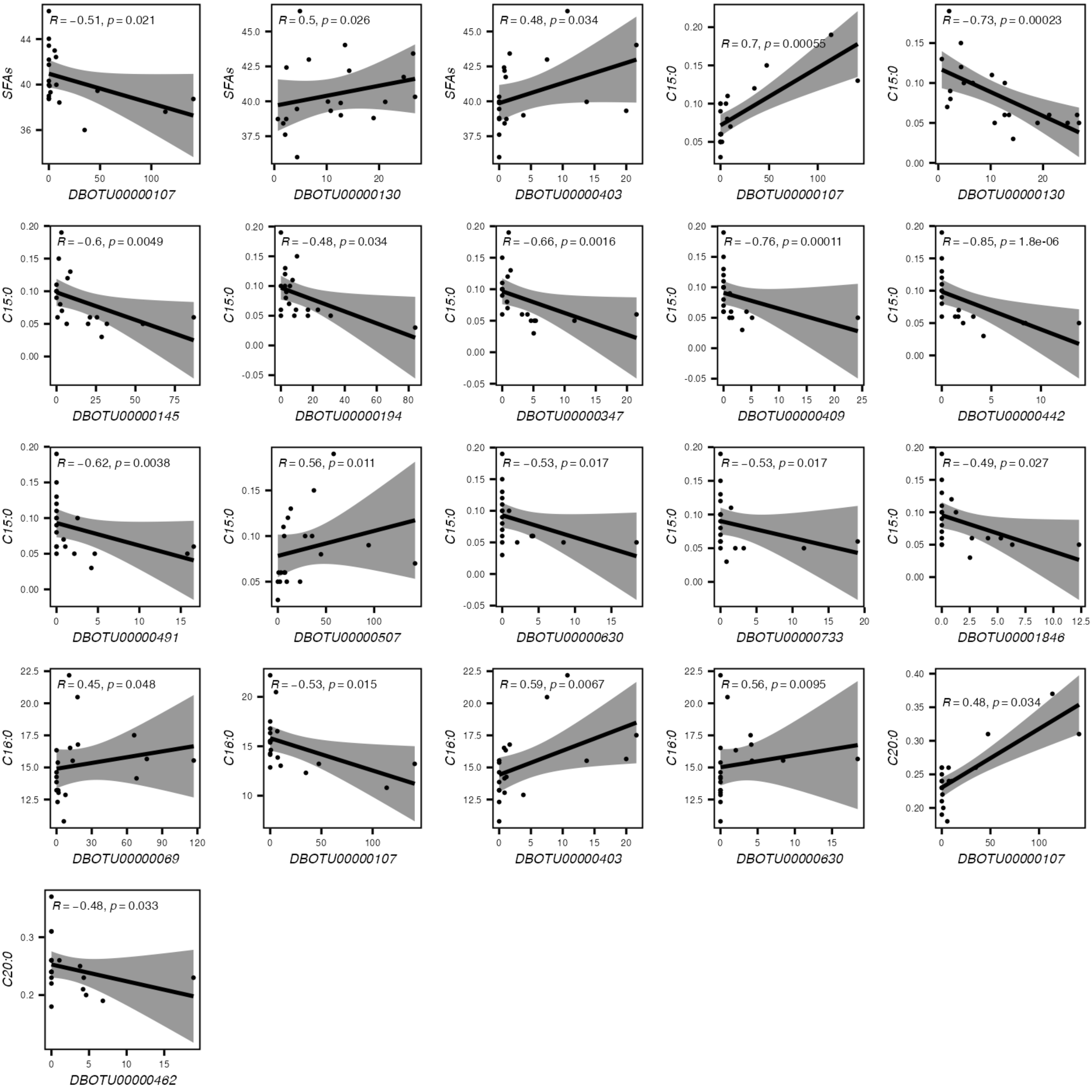

Supplementary Figure 5

Supplementary Figure 5. Spearman linear correlations on corrected values for OTUs after DESeq2 normalization (X axis) and the retinal amount of SFAs (Y axis), showing significant correlations among both datasets. n=10/group.

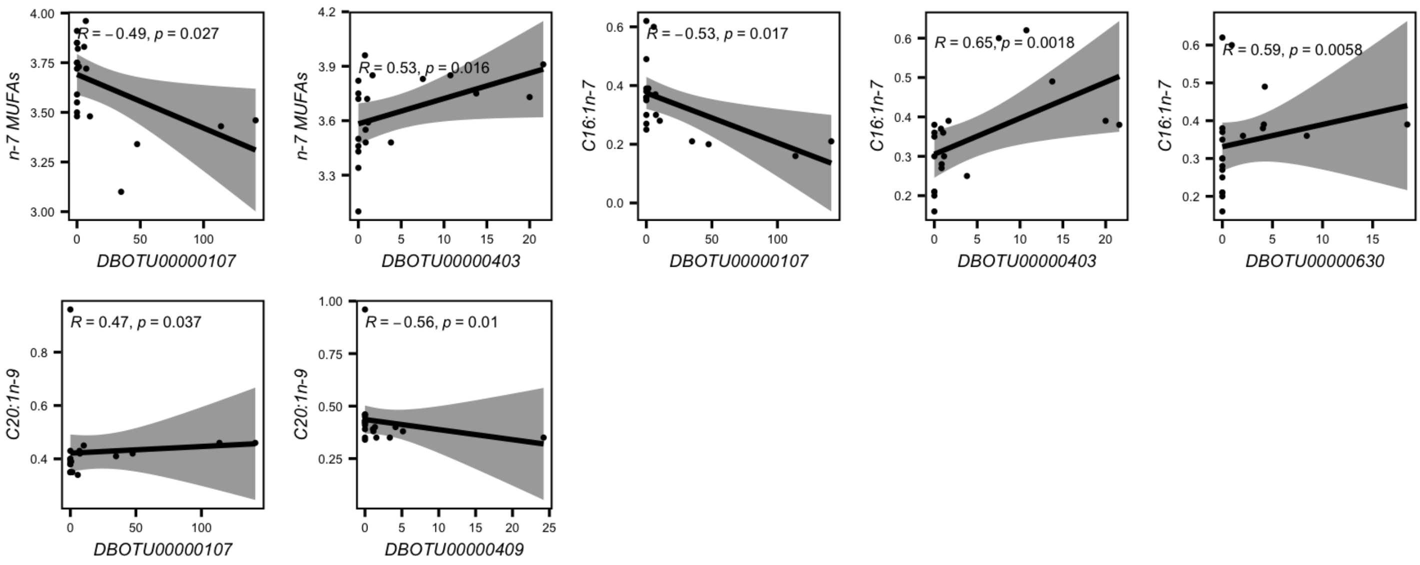

Supplementary Figure 6

Supplementary Figure 6. Spearman linear correlations on corrected values for OTUs after DESeq2 normalization (X axis) and the retinal amount of MUFAs (Y axis), showing significant correlations among both datasets. n=10/group.

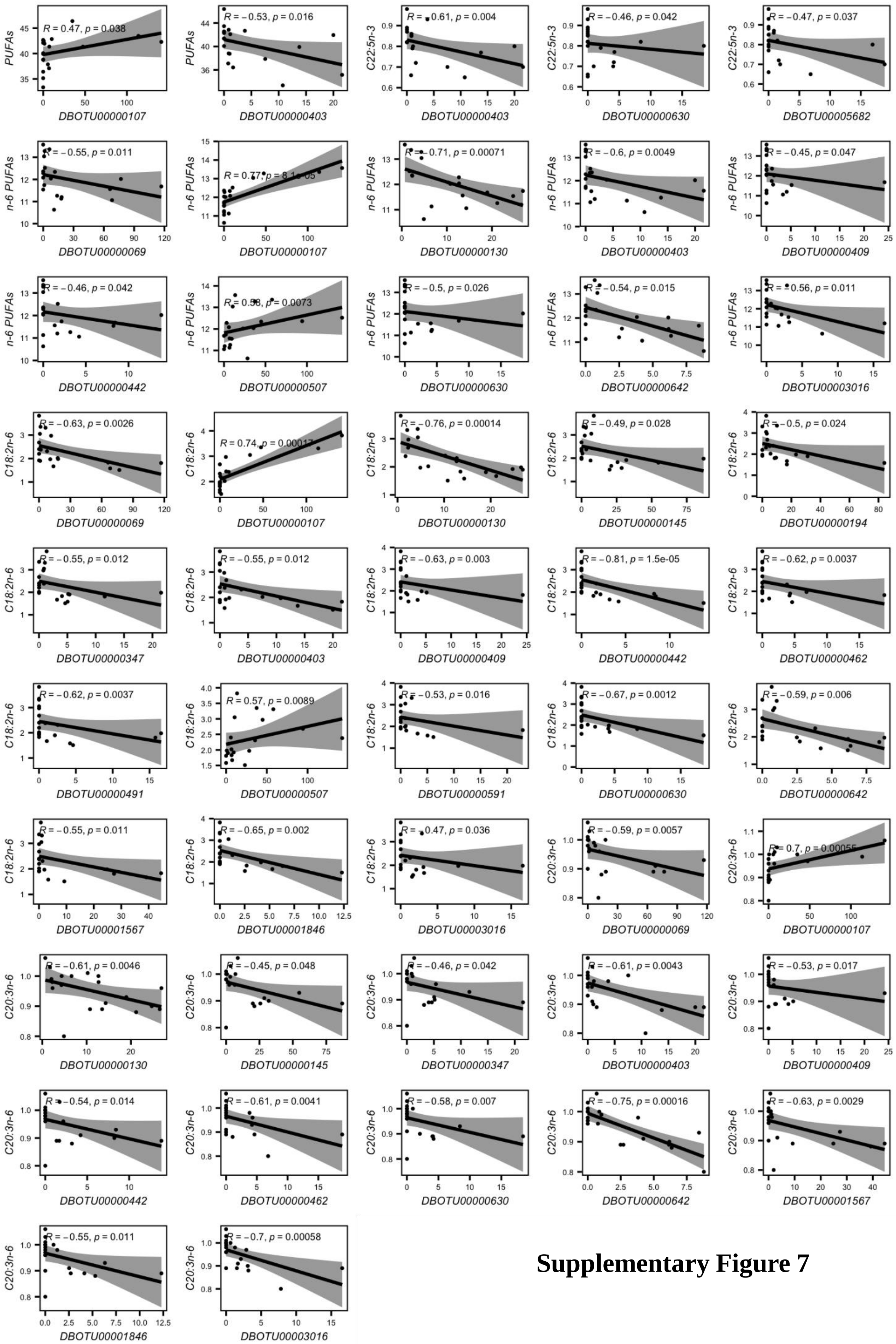

Supplementary Figure 7

Supplementary Figure 7. Spearman linear correlations on corrected values for OTUs after DESeq2 normalization (X axis) and the retinal amount of PUFAs (Y axis), showing significant correlations among both datasets. n=10/group.

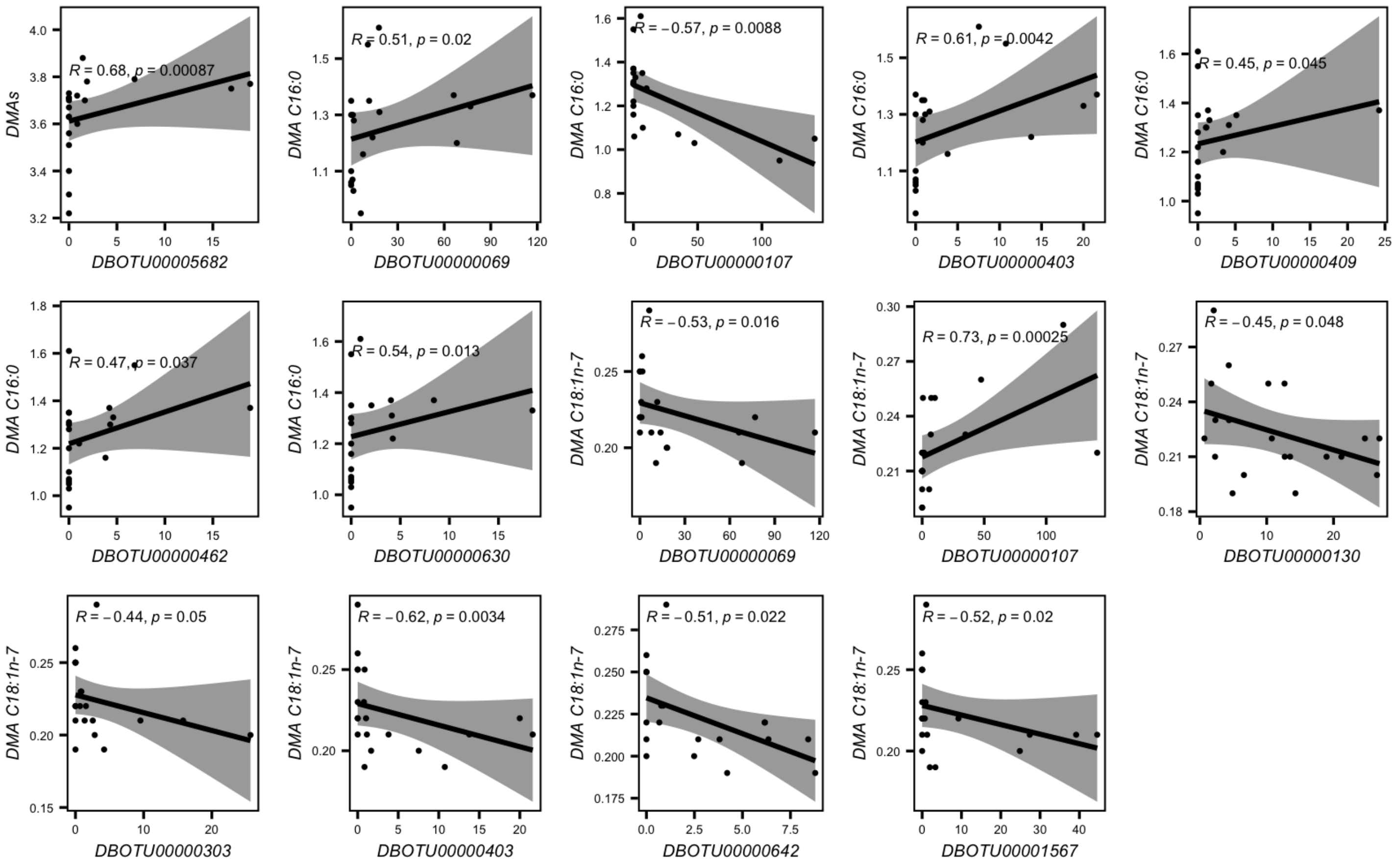

Supplementary Figure 8

Supplementary Figure 8. Spearman linear correlations on corrected values for OTUs after DESeq2 normalization (X axis) and the retinal amount of DMAs (Y axis), showing significant correlations among both datasets. n=10/group.

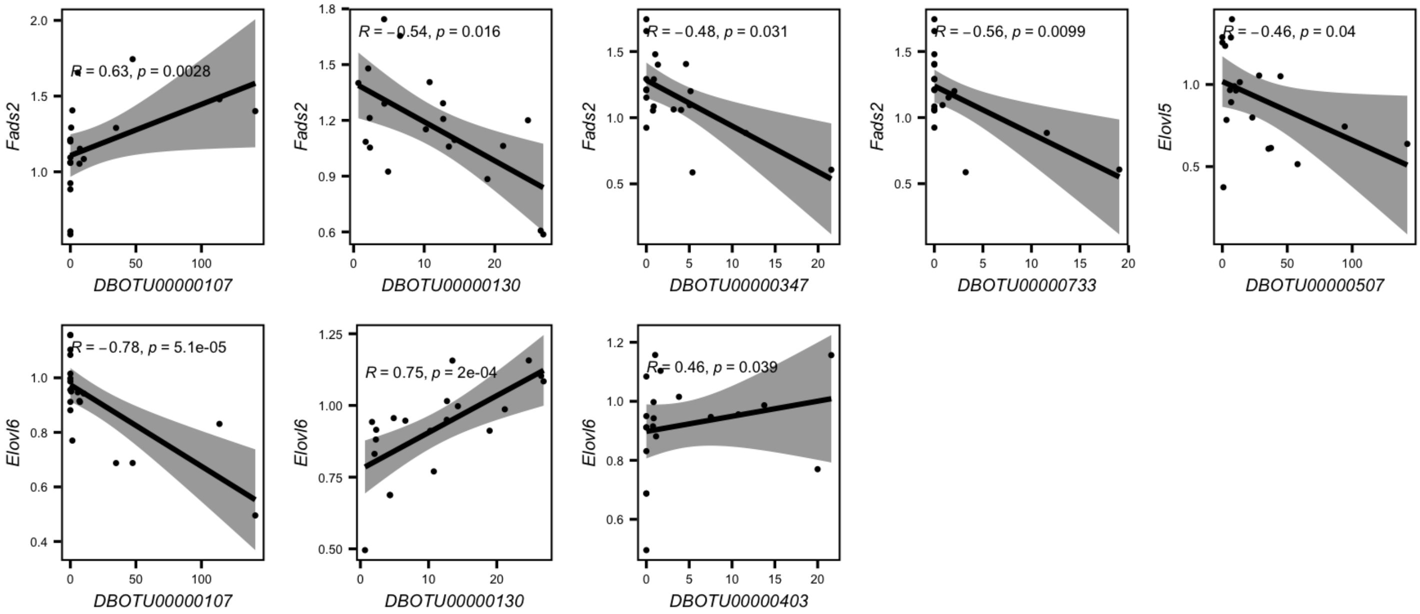

Supplementary Figure 9

Supplementary Figure 9. Spearman linear correlations on corrected values for OTUs after DESeq2 normalization (X axis) and the retinal expression level of elongases and desaturases (Y axis), showing significant correlations among both datasets. n=10/group.

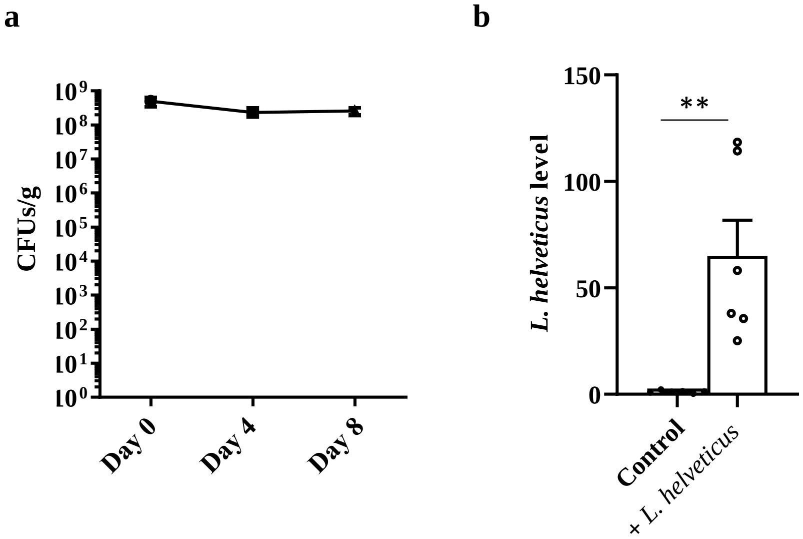

Supplementary Figure 10

Supplementary Figure 10. Quantification of *L. helveticus* in mouse food and in mouse fecal samples. (**a**) Viability of *L. helveticus* following its incorporation into the diet and its storage at 4°C. The probiotic was incorporated into the diet at the concentration of 1.10^9 CFUs of *L. helveticus*/g of food. Portions were molded using Petri dishes (20g/dish) and stored at 4°C under anaerobic conditions. The number of viable CFUs of *L. helveticus*/portion was determined by resuspending the diet, plating dilutions of the suspension on MRS agar plates and enumerating the number of CFUs. Results are expressed as CFUs/g of diet at day 0 (the day the food containing *L. helveticus* was prepared), and into portions stored during 4 days or 8 days at 4°C under anaerobic conditions. n=3/day. (**b**) Quantification of *L. helveticus* DNA in mouse fecal samples by qPCR. The DNA amount of *L. helveticus* was normalized to that of total eubacteria. Results are expressed as fold change. Quantification was performed on fresh feces of 6 different mice in each mouse group. Data are presented as mean ± s.e.m.. Mann-Whitney test (** $p<0.01$).

Supplementary Tables

Supplementary Table 1. Fatty acid composition in the liver of mice fed a control diet or a diet supplemented with *L. helveticus*.

	**Control**	**+ *L. helveticus***
C14:0	0.472 ± 0.014	0.438 ± 0.015
C15:0	0.110 ± 0.005	0.117 ± 0.004
C16:0**	24.019 ± 0.238	22.893 ± 0.205
C17:0	0.122 ± 0.004	0.122 ± 0.005
C18:0	4.023 ± 0.250	3.786 ± 0.145
C20:0	0.121 ± 0.009	0.128 ± 0.015
**Total SFAs****	**28.868 ± 0.292**	**27.484 ± 0.307**
C16:1n-7	6.153 ± 0.210	6.555 ± 0.357
C18:1n-7	5.348 ± 0.456	4.586 ± 0.275
**Total MUFAs n-7**	**11.501 ± 0.496**	**11.141 ± 0.461**
C16:1n-9	1.027 ± 0.093	0.962 ± 0.051
C18:1n-9	32.696 ± 1.580	31.571 ± 0.917
C20:1n-9	0.559 ± 0.055	0.499 ± 0.034
C22:1n-9	0.060 ± 0.002	0.067 ± 0.007
**Total MUFAs n-9**	**34.341 ± 1.726**	**33.099 ± 0.997**
C18:1t	0.070 ± 0.006	0.076 ± 0.011
**Total MUFAs**	**45.912 ± 2.142**	**44.316 ± 1.061**
C18:3n-3	0.281 ± 0.041	0.255 ± 0.026
C20:5n-3	0.323 ± 0.032	0.400 ± 0.017
C22:5n-3	0.308 ± 0.030	0.348 ± 0.013
C22:6n-3	4.058 ± 0.383	4.344 ± 0.154
**Total PUFAs n-3**	**4.970 ± 0.458**	**5.347 ± 0.194**
C18:2n-6	14.339 ± 1.161	16.748 ± 0.714
C18:3n-6	0.332 ± 0.058	0.413 ± 0.065
C20:2n-6	0.131 ± 0.004	0.141 ± 0.004
C20:3n-6	0.172 ± 0.009	0.157 ± 0.011
C20:4n-6	4.918 ± 0.425	4.934 ± 0.164
C22:4n-6	0.167 ± 0.013	0.166 ± 0.009
C22:5n-6	0.101 ± 0.012	0.095 ± 0.008
**Total PUFAs n-6**	**20.160 ± 1.619**	**22.654 ± 0.894**
**Total PUFAs**	**25.130 ± 2.065**	**28.001 ± 1.078**
**n-6/n-3 ratio**	**4.102 ± 0.099**	**4.236 ± 0.057**
DMA C18:1n-9	0.201 ± 0.009	0.199 ± 0.005

Results are expressed as percentages of total fatty acid methyl esters (FAMEs) + dimethylacetals (DMAs). SFAs: saturated fatty acids. MUFAs: monounsaturated fatty acids. PUFAs: polyunsaturated fatty acids. Control group, n=9. *L. helveticus* group, n=10. Data are mean ± s.e.m.. Mann-Whitney test. ***p*<0.01.

Supplementary Table 2. Fatty acid composition in the plasma of mice fed a control diet or a diet supplemented with *L. helveticus*.

	**Control**	**+ *L. helveticus***
C14:0*	0.048 ± 0.008	0.029 ± 0.006
C15:0	0.057 ± 0.005	0.044 ± 0.005
C16:0*	15.784 ± 0.726	13.447 ± 0.429
C17:0	0.186 ± 0.004	0.185 ± 0.008
C18:0	8.142 ± 0.156	7.938 ± 0.176
C20:0*	0.193 ± 0.008	0.232 ± 0.012
C22:0*	0.065 ± 0.004	0.090 ± 0.009
C24:0	0.084 ± 0.006	0.102 ± 0.006
**Total SFAs****	**24.559 ± 0.668**	**22.067 ± 0.402**
C16:1n-7	2.509 ± 0.207	2.161 ± 0.167
C18:1n-7	3.249 ± 0.133	3.133 ± 0.146
C20:1n-7	0.179 ± 0.009	0.206 ± 0.011
**Total MUFAs n-7**	**5.938 ± 0.286**	**5.500 ± 0.301**
C16:1n-9*	0.279 ± 0.027	0.199 ± 0.015
C18:1n-9 (p=0.506)	16.401 ± 0.262	15.615 ± 0.260
C20:1n-9	0.422 ± 0.017	0.476 ± 0.030
C22:1n-9**	0.584 ± 0.047	0.856 ± 0.092
C24:1n-9	0.148 ± 0.009	0.174 ± 0.010
**Total MUFAs n-9**	**17.835 ± 0.260**	**17.321 ± 0.271**
C18:1t**	0.157 ± 0.008	0.201 ± 0.014
**Total MUFAs**	**23.930 ± 0.521**	**23.022 ± 0.446**
C18:3n-3	0.273 ± 0.011	0.280 ± 0.008
C20:5n-3	0.628 ± 0.034	0.690 ± 0.019
C22:5n-3	0.344 ± 0.018	0.385 ± 0.016
C22:6n-3 (p=0.0653)	7.797 ± 0.404	8.605 ± 0.246
**Total PUFAs n-3***	**9.042 ± 0.430**	**9.960 ± 0.256**
C18:2n-6	19.081 ± 0.757	20.751 ± 0.545
C18:3n-6	0.337 ± 0.038	0.380 ± 0.039
C20:2n-6*	0.135 ± 0.006	0.149 ± 0.004
C20:3n-6	1.662 ± 0.105	1.613 ± 0.067
C20:4n-6	20.010 ± 0.661	20.776 ± 0.575
C22:4n-6	0.119 ± 0.004	0.131 ± 0.007
C22:5n-6	0.076 ± 0.011	0.082 ± 0.004
**Total PUFAs n-6***	**41.420 ± 0.735**	**43.882 ± 0.541**
**n-6/n-3 ratio**	**4.647 ± 0.159**	**4.422 ± 0.088**
C20:3n-9	0.575 ± 0.060	0.593 ± 0.035
**Total PUFAs***	**51.036 ± 1.087**	**54.435 ± 0.709**
DMA C16:0*	0.142 ± 0.013	0.112 ± 0.008
DMA C18:0*	0.168 ± 0.009	0.204 ± 0.012
DMA C18:1n-7	0.040 ± 0.002	0.042 ± 0.001
DMA C18:1n-9	0.125 ± 0.004	0.118 ± 0.004
**Total DMAs**	**0.475 ± 0.021**	**0.476 ± 0.012**

Results are expressed as percentages of total fatty acid methyl esters (FAMEs) + dimethylacetals (DMAs). SFAs: saturated fatty acids. MUFAs: monounsaturated fatty acids.

PUFAs: polyunsaturated fatty acids. Control group, n=10. *L. helveticus* group, n=9. Data are mean ± s.e.m.. Mann-Whitney test. *p<0.05 and **p<0.01.

Supplementary Table 3. Fatty acid composition in the retina of mice fed a control diet or a diet supplemented with *L. helveticus*.

	**Control**	**+ *L. helveticus***
C14:0	0.081 ± 0.017	0.068 ± 0.008
C15:0**	0.061 ± 0.007	0.110 ± 0.012
C16:0***	16.954 ± 0.793	13.470 ± 0.478
C17:0	0.155 ± 0.007	0.142 ± 0.007
C18:0	24.449 ± 0.546	24.962 ± 0.273
C20:0*	0.223 ± 0.009	0.270 ± 0.015
**Total SFAs****	**41.923 ± 0.756**	**39.022 ± 0.528**
C16:1n-7**	0.421 ± 0.036	0.266 ± 0.023
C18:1n-7	3.323 ± 0.037	3.272 ± 0.060
**Total MUFAs n-7***	**3.744 ± 0.042**	**3.538 ± 0.078**
C16:1n-9*	0.186 ± 0.017	0.145 ± 0.008
C18:1n-9	11.024 ± 0.166	10.624 ± 0.171
C20:1n-9**	0.427 ± 0.060	0.425 ± 0.009
C22:1n-9	0.376 ± 0.032	0.368 ± 0.043
**Total MUFAs n-9**	**12.013 ± 0.196**	**11.562 ± 0.196**
C18:1t	0.119 ± 0.007	0.118 ± 0.008
**Total MUFAs** (p=0.0630)	**15.876 ± 0.225**	**15.218 ± 0.238**
C18:3n-3	0.191 ± 0.009	0.184 ± 0.016
C20:5n-3	0.359 ± 0.012	0.349 ± 0.015
C22:5n-3**	0.762 ± 0.021	0.851 ± 0.027
C22:6n-3*	25.795 ± 0.813	28.135 ± 0.672
**Total PUFAs n-3***	**27.107 ± 0.829**	**29.519 ± 0.690**
C18:2n-6****	1.819 ± 0.056	2.847 ± 0.171
C18:3n-6	0.069 ± 0.006	0.141 ± 0.035
C20:2n-6	0.370 ± 0.040	0.379 ± 0.014
C20:3n-6***	0.905 ± 0.017	0.998 ± 0.009
C20:4n-6	7.299 ± 0.141	7.343 ± 0.058
C22:4n-6	0.814 ± 0.024	0.870 ± 0.016
C22:5n-6	0.106 ± 0.013	0.109 ± 0.018
**Total PUFAs n-6******	**11.382 ± 0.127**	**12.687 ± 0.180**
**n-6/n-3 ratio**	**0.423 ± 0.011**	**0.432 ± 0.012**
**Total PUFAs****	**38.489 ± 0.916**	**42.206 ± 0.725**
DMA C16:0**	1.361 ± 0.041	1.135 ± 0.042
DMA C18:0	1.896 ± 0.026	1.942 ± 0.062
DMA C18:1n-7**	0.207 ± 0.004	0.240 ± 0.008
DMA C18:1n-9	0.249 ± 0.006	0.246 ± 0.006
**Total DMAs***	**3.713 ± 0.023**	**3.563 ± 0.065**

Results are expressed as percentages of total fatty acid methyl esters (FAMEs) + dimethylacetals (DMAs). SFAs: saturated fatty acids. MUFAs: monounsaturated fatty acids. PUFAs: polyunsaturated fatty acids. n=10/group. Data are mean ± s.e.m.. Mann-Whitney test. *p<0.05, **p<0.01, ***p<0.001 and ****p<0.0001.

Supplementary Table 4. Relative amounts of phospholipid species in the retina.

	Control	+ *L. helveticus*
**Ethanolanine glycerophospholipids**		
Phosphatidylethanolamine (PE)		
PE(16:0/16:0)	0.252 ± 0.012	0.241 ± 0.006
PE(16:0/18:0)	0.370 ± 0.016	0.351 ± 0.010
PE(16:0/18:1)	2.122 ± 0.094	2.197 ± 0.053
PE(16:0/20:4)	1.274 ± 0.052	1.230 ± 0.022
PE(16:0/20:5)	0.364 ± 0.007	0.359 ± 0.008
PE(16:0/22:6)*	14.769 ± 0.113	15.334 ± 0.204
PE(16:1/22:6)*	0.208 ± 0.004	0.225 ± 0.005
PE(18:0/18:1)	2.465 ± 0.106	2.586 ± 0.059
PE(18:0/22:4)	0.646 ± 0.064	0.760 ± 0.009
PE(18:0/22:6)	26.065 ± 0.427	25.647 ± 0.500
PE(18:1/18:1)**	0.602 ± 0.053	0.730 ± 0.015
PE(18:1/18:2)**	0.401 ± 0.017	0.481 ± 0.015
PE(18:1/22:6)	3.614 ± 0.068	3.637 ± 0.101
PE(18:2/22:6)	0.275 ± 0.008	0.307 ± 0.017
PE(20:0/22:6)	0.183 ± 0.003	0.184 ± 0.004
PE(20:1/22:6)	0.350 ± 0.006	0.356 ± 0.010
PE(20:2/22:6)	0.255 ± 0.009	0.237 ± 0.012
PE(20:3/22:6)	0.485 ± 0.021	0.419 ± 0.043
PE(20:4/22:6)	0.285 ± 0.015	0.253 ± 0.010
PE(22:4/22:6)	0.330 ± 0.015	0.334 ± 0.010
PE(22:5/22:6)	1.258 ± 0.064	1.205 ± 0.051
PE(22:6/22:6)	6.643 ± 0.380	6.139 ± 0.278
PE(24:5/22:6)	0.205 ± 0.013	0.203 ± 0.009
PE(24:6/22:6)	0.554 ± 0.039	0.560 ± 0.031
PE(18:0/20:4); PE(18:1/20:3)	5.769 ± 0.293	5.396 ± 0.100
PE(18:0/20:5); PE(18:1/20:4)*	2.362 ± 0.037	2.472 ± 0.045
Plasmenylethanolamine (PlE)		
PE(P-16:0/16:0)	0.187 ± 0.009	0.194 ± 0.008
PE(P-16:0/18:1)*	1.047 ± 0.035	1.137 ± 0.042
PE(P-16:0/18:2)**	0.244 ± 0.011	0.306 ± 0.018
PE(P-16:0/20:3)*	0.552 ± 0.009	0.588 ± 0.017
PE(P-16:0/20:4) (*p*=0.0654)	3.521 ± 0.080	3.269 ± 0.068
PE(P-16:0/20:5)	0.252 ± 0.018	0.257 ± 0.014
PE(P-18:0/16:0)	0.675 ± 0.029	0.682 ± 0.023
PE(P-18:0/18:1)*	0.265 ± 0.009	0.280 ± 0.007
PE(P-18:0/18:2)*	0.196 ± 0.006	0.224 ± 0.007
PE(P-18:0/20:3)	0.450 ± 0.008	0.445 ± 0.008
PE(P-18:0/20:4)	3.774 ± 0.079	3.608 ± 0.074
PE(P-18:1/22:6)	0.801 ± 0.034	0.811 ± 0.023
PE(P-20:0/20:3)	0.173 ± 0.004	0.171 ± 0.004
PE(P-16:0/22:6); PE(P-18:1/20:5)	4.972 ± 0.132	5.174 ± 0.129
PE(O-16:0/22:6)§; PE(P-18:1/20:4)	2.600 ± 0.041	2.676 ± 0.075
PE(P-18:0/22:4); PE (P-20:0/20:4)	0.680 ± 0.081	0.701 ± 0.011
PE(P-18:0/22:6); PE(P-18:1/22:5)	6.357 ± 0.210	6.232 ± 0.150
PE(O-18:0/22:6)§; PE(P-20:0/20:5)	1.144 ± 0.164	1.401 ± 0.042

**Choline glycerophospholipids**		
Phosphatidylcholine (PC)		
PC(14:0/16:0)*	0.881 ± 0.046	0.981 ± 0.029
PC(16:0/16:0)	17.509 ± 0.506	18.176 ± 0.398
PC(16:0/16:1)*	2.194 ± 0.062	2.413 ± 0.062
PC(16:0/18:0)	4.028 ± 0.158	4.147 ± 0.093
PC(16:0/18:1)	17.869 ± 0.428	17.914 ± 0.363
PC(16:0/18:2); PC(16:1/18:1)*	1.223 ± 0.026	1.344 ± 0.042
PC(16:0/18:3)	0.565 ± 0.044	0.621 ± 0.046
PC(16:0/20:3); PC(18:1/18:2)**	0.813 ± 0.012	0.859 ± 0.014
PC(16:0/20:4)	2.445 ± 0.079	2.393 ± 0.048
PC(16:0/20:5)	0.159 ± 0.004	0.161 ± 0.003
PC(16:0/22:6)	10.252 ± 0.301	10.089 ± 0.261
PC(16:1/22:6)	0.226 ± 0.012	0.235 ± 0.013
PC(18:0/18:0)	0.747 ± 0.029	0.747 ± 0.031
PC(18:0/18:1)	6.765 ± 0.199	6.804 ± 0.211
PC(18:0/20:2)	0.163 ± 0.006	0.175 ± 0.006
PC(18:0/20:4); PC(16:0/22:4)	3.284 ± 0.081	3.214 ± 0.051
PC(18:0/22:6)	13.748 ± 0.435	13.518 ± 0.338
PC(18:1/18:1)	1.931 ± 0.049	2.007 ± 0.053
PC(18:1/20:4)	1.423 ± 0.041	1.385 ± 0.036
PC(18:1:22:6)	1.437 ± 0.054	1.137 ± 0.171
PC(20:0/22:6)	0.159 ± 0.004	0.156 ± 0.005
PC(20:1/22:6)	0.142 ± 0.005	0.144 ± 0.006
PC(20:3/22:6)	0.627 ± 0.062	0.646 ± 0.061
PC(20:4/22:6); PC(20:5/22:5)	0.210 ± 0.025	0.217 ± 0.012
PC(22:6/22:6)	3.111 ± 0.204	2.674 ± 0.188
PC(24:6/22:6)	0.229 ± 0.014	0.203 ± 0.014
PC(32:5/22:6)	0.384 ± 0.021	0.340 ± 0.022
PC(32:6/22:6)	1.244 ± 0.069	1.108 ± 0.078
PC(34:5/22:6)	0.640 ± 0.038	0.622 ± 0.043
PC(34:6/22:6)	1.621 ± 0.101	1.589 ± 0.119
PC(36:6/22:6)	0.196 ± 0.017	0.224 ± 0.024
PC(37:6)	0.202 ± 0.011	0.214 ± 0.018
PC(38:3)	0.529 ± 0.007	0.543 ± 0.007
PC(40:5)	2.000 ± 0.064	1.967 ± 0.050
PC(40:9)	0.315 ± 0.036	0.317 ± 0.034
Plasmenylcholine (PlC)		
PC(P-16:0/16:0)	0.172 ± 0.011	0.176 ± 0.007
PC(P-18:0/16:0)	0.182 ± 0.009	0.172 ± 0.018
PC(P-18:1/16:0); PC(P-16:0/18:1)	0.373 ± 0.014	0.369 ± 0.019
**Serine glycerophospholipids**		
Phosphatidylserine (PS)		
PS(16:0/20:4)	1.543 ± 0.164	1.747 ± 0.103
PS(18:0/20:4)	3.685 ± 0.402	3.244 ± 0.279
PS(18:0/22:6)	11.832 ± 1.764	9.329 ± 0.883
PS (22:6/22:6)	7.100 ± 1.708	4.180 ± 0.545
PS(37:3)	2.417 ± 0.260	2.726 ± 0.140
PS(37:4) (*p*=0.0524)	1.697 ± 0.196	2.186 ± 0.123

PS(37:5)	4.039 ± 0.363	4.748 ± 0.325
PS(39:5)	5.941 ± 0.526	6.353 ± 0.446
PS(39:6)	2.697 ± 0.277	3.066 ± 0.209
PS(40:3)	7.338 ± 0.684	8.218 ± 0.336
PS(42:3)	11.845 ± 0.510	12.244 ± 0.381
PS(42:5)	38.492 ± 1.571	40.979 ± 1.098
Plasmenylserine (PlS)		
PS(P-16:0/20:4)	1.373 ± 0.124	0.980 ± 0.218
**Inositol glycerophospholipids**		
Phosphatidylinositol (PI)		
PI(16:0/16:0)	0.061 ± 0.009	0.070 ± 0.004
PI(16:0/18:0)	0.111 ± 0.007	0.124 ± 0.007
PI(16:0/18:1)	0.684 ± 0.030	0.742 ± 0.049
PI(16:0/18:2)*	0.259 ± 0.010	0.310 ± 0.018
PI(16:0/20:3)	2.833 ± 0.063	2.889 ± 0.045
PI(16:0/20:4)	18.659 ± 0.234	18.911 ± 0.179
PI(16:0/20:5)*	1.314 ± 0.036	1.441 ± 0.064
PI(16:0/22:6)	3.110 ± 0.062	3.104 ± 0.122
PI(17:0/20:4)	0.187 ± 0.003	0.199 ± 0.013
PI(18:0/20:3)	8.800 ± 0.072	8.658 ± 0.050
PI(18:0/20:4)*	53.580 ± 0.466	52.303 ± 0.318
PI(18:0/22:6)	1.442 ± 0.036	1.375 ± 0.044
PI(18:1/22:6)	0.519 ± 0.015	0.506 ± 0.021
PI(18:1/20:4); PI(18:0/20:5)**	7.126 ± 0.622	8.090 ± 0.071
PI(20:4/22:6)	0.355 ± 0.015	0.319 ± 0.014
PI(22:6/22:6)	0.059 ± 0.007	0.063 ± 0.005
PI(36:1)	0.251 ± 0.011	0.257 ± 0.011
PI(36:2)	0.275 ± 0.013	0.309 ± 0.018
PI(40:5)	0.374 ± 0.007	0.330 ± 0.032
**Sphingomyelins (SM)**		
SM(d18:0/16:0)	3.251 ± 0.038	3.302 ± 0.034
SM(d18:0/18:0)	2.970 ± 0.050	3.014 ± 0.036
SM(d18:1/16:0)	33.164 ± 0.438	33.836 ± 0.352
SM(d18:1/18:0)	27.862 ± 0.454	28.319 ± 0.408
SM(d18:1/18:1)	2.686 ± 0.070	2.565 ± 0.143
SM(d18:1/20:0)	6.634 ± 0.181	6.366 ± 0.150
SM(d18:1/20:1)	1.564 ± 0.105	1.705 ± 0.257
SM(d18:1/22:0)	2.935 ± 0.187	2.621 ± 0.156
SM(d18:1/22:1)	1.092 ± 0.074	0.892 ± 0.119
SM(d18:1/24:0)	1.252 ± 0.089	0.881 ± 0.213
SM(d18:1/24:1)	2.664 ± 0.100	2.629 ± 0.102
SM(d18:2/18:1)	6.769 ± 0.335	6.809 ± 0.313
SM(d18:2/20:1)	5.915 ± 0.285	5.954 ± 0.229
SM(d18:2/22:1)	1.241 ± 0.121	1.108 ± 0.134

Lipid species were analyzed by LC-MS^2. For each phospholipid class, results are expressed as abundance (in percentage) of each species relative to that of total species, defined as 100%. It should be noted that for some isobaric phospholipid species, the different possible combinations

of fatty acids moiety position are presented. § Plasmanylethanolamine species. n=10/group. Data are mean ± s.e.m.. Mann-Whitney test. *p<0.05 and **p<0.01.

Supplementary Table 5. Fatty acid profiles of diets.

	Not supplemented	*+ L. helveticus*
C14:0	0.477 ± 0.006	0.460 ± 0.010
C15:0	0.183 ± 0.006	0.180 ± 0.000
C16:0	20.510 ± 0.026	20.150 ± 0.170
C17:0	0.133 ± 0.006	0.130 ± 0.000
C18:0	2.270 ± 0.017	2.240 ± 0.000
C20:0	0.290 ± 0.010	0.293 ± 0.006
C22:0	0.193 ± 0.012	0.183 ± 0.006
C24:0	0.203 ± 0.006	0.200 ± 0.010
**Total SFAs**	**24.260 ± 0.026**	**23.837 ± 0.172**
C16:1n-7	0.510 ± 0.000	0.503 ± 0.012
C18:1n-7	1.230 ± 0.026	1.250 ± 0.010
**Total MUFAs n-7**	**1.740 ± 0.026**	**1.753 ± 0.006**
C16:1n-9	0.100 ± 0.000	0.097 ± 0.006
C18:1n-9	20.380 ± 0.056	19.917 ± 0.060
C20:1n-9	0.767 ± 0.006	0.753 ± 0.015
C22:1n-9	0.090 ± 0.000	0.090 ± 0.010
**Total MUFAs n-9**	**21.337 ± 0.055**	**20.857 ± 0.074**
**Total MUFAs**	**23.077 ± 0.032**	**22.610 ± 0.072**
C18:3n-3	3.307 ± 0.012	3.397 ± 0.025
C20:5n-3	0.300 ± 0.000	0.297 ± 0.006
C22:5n-3	0.160 ± 0.010	0.157 ± 0.006
C22:6n-3	0.497 ± 0.012	0.503 ± 0.012
**Total PUFAs n-3**	**4.263 ± 0.021**	**4.353 ± 0.023**
C18:2n-6	48.173 ± 0.042	48.987 ± 0.093
C20:2n-6	0.130 ± 0.017	0.120 ± 0.000
C20:4n-6	0.097 ± 0.006	0.093 ± 0.006
**Total PUFAs n-6**	**48.400 ± 0.046**	**49.200 ± 0.095**
**Ratio n-6/n-3**	**11.353 ± 0.065**	**11.302 ± 0.054**
**Total PUFAs**	**52.663 ± 0.029**	**53.553 ± 0.108**

Results are expressed as percentages of total fatty acid methyl esters (FAMEs). SFAs: saturated fatty acids. MUFAs: monounsaturated fatty acids. PUFAs: polyunsaturated fatty acids. n=3/diet. Data are mean ± s.d..

Supplementary Table 6. Primers used in this study.

Gene	Forward primer (5′→3′)	Reverse primer (5′→3′)
Fads1	CGCCAAACGCGCTACTTTAC	CCACAAAAGGATCCGTGGCA
Fads2	CGTGGGCAAGTTCTTGAAGC	TCTGAGAGCTTTTGCCACGG
Scd1	CAGGAGGGCAGGTTTCCAAG	CGTTCATTTCCGGAGGGAGG
Elovl1	CCTGAAGCACTTCGGATGGT	TCACTTGCCCGTCCTTCTTC
Elovl2	GTGATGTCCGGGTAGCCAAG	GGACGCGTGGTGATAGACAT
Elovl3	TACTTCTTTGGCTCTCGCCC	AGCTTACCCAGTACTCCTCCA
Elovl4	TGAAGTCAGGATAGCTGGCG	AGTGAACATGGTGCAGTGGT
Elovl5	TGATGAACTGGGTTCCCTGC	CAGCTGCCCTTGAGTGATGT
Elovl6	AGAACACGTAGCGACTCCGA	TCAGATGCCGACCACCAAAG
Fasn	GACTCGGCTACTGACACGAC	CGAGTTGAGCTGGGTTAGGG
Far1	GCTCGGAAGCATCTCAACAAG	GTGCTGGATGCTCGGAAGTAT
Gnpat	TCACCGCAGCTACATTGACT	GCAGCTCACTGACCACTCTC
Agps	GTGCAGGGTGACACAGACTT	CCATGGTGATGTGACAGGCT
Hprt	CAGTCCCAGCGTCGTGATTA	TGGCCTCCCATCTCCTTCAT
